# Development of a simple and versatile *in vitro* method for production, stimulation, and analysis of bioengineered muscle

**DOI:** 10.1371/journal.pone.0272610

**Published:** 2022-08-11

**Authors:** Karen Wells-Cembrano, Júlia Sala-Jarque, Jose A. del Rio

**Affiliations:** 1 Molecular and Cellular Neurobiotechnology, Institute for Bioengineering of Catalonia (IBEC), Scientific Park of Barcelona, The Barcelona Institute for Science and Technology (BIST), Barcelona, Spain; 2 Department of Cell Biology, Physiology and Immunology, University of Barcelona, Barcelona, Spain; 3 Network Centre of Biomedical Research of Neurodegenerative Diseases (CIBERNED), Institute of Health Carlos III, Ministry of Economy and Competitiveness, Spain; 4 Institute of Neuroscience, University of Barcelona, Barcelona, Spain; University of Minnesota Medical School, UNITED STATES

## Abstract

In recent years, 3D *in vitro* modeling of human skeletal muscle has emerged as a subject of increasing interest, due to its applicability in basic studies or screening platforms. These models strive to recapitulate key features of muscle architecture and function, such as cell alignment, maturation, and contractility in response to different stimuli. To this end, it is required to culture cells in biomimetic hydrogels suspended between two anchors. Currently available protocols are often complex to produce, have a high rate of breakage, or are not adapted to imaging and stimulation. Therefore, we sought to develop a simplified and reliable protocol, which still enabled versatility in the study of muscle function. In our method, we have used human immortalized myoblasts cultured in a hydrogel composed of Matrigel^TM^ and fibrinogen, to create muscle strips suspended between two VELCRO^TM^ anchors. The resulting muscle constructs show a differentiated phenotype and contractile activity in response to electrical, chemical and optical stimulation. This activity is analyzed by two alternative methods, namely contraction analysis and calcium analysis with Fluo-4 AM. In all, our protocol provides an optimized version of previously published methods, enabling individual imaging of muscle bundles and straightforward analysis of muscle response with standard image analysis software. This system provides a start-to-finish guide on how to produce, validate, stimulate, and analyze bioengineered muscle. This ensures that the system can be quickly established by researchers with varying degrees of expertise, while maintaining reliability and similarity to native muscle.

## Introduction

The development of 3D *in vitro* models of skeletal muscle has become a subject of increasing interest, with publications steadily growing in the past 10 years [1, 2]. These culture models are useful for studying muscle function in health and disease, or as screening platforms for clinical research. In order to mimic *in vivo* muscle physiology, they must recapitulate relevant features of skeletal muscle architecture and function, such as the formation of aligned and compacted muscle fibers, and contractile ability in response to electrical or chemical stimulation [3].

In order to obtain differentiated skeletal muscle cells, some models use primary myoblasts [4, 5] due to their close reproduction of *in vivo* phenotype; but this requires having a source for extraction of primary myoblasts, which increases animal experimentation. To solve this issue and adhere to the 3R strategy of animal experimentation, numerous immortalized myoblast cell lines have been generated, which can be used in *in vitro* platforms. One of the most used cell lines is the C2C12 mouse myoblast line [6], which has been extensively characterized and serves as a robust model of mouse skeletal muscle function [7, 8]. Nevertheless, human myoblasts are also needed for more accurate modeling of human skeletal muscle. Several immortalized human myoblast cell lines have been generated in recent years [9–11], from both healthy samples and neuromuscular disease patients.

Concerning culture type, as it occurs with many other tissues and organs, emerging 3D tissue engineering techniques have proven to be a more accurate way of modeling native tissue architecture and function in skeletal muscle. Using 2D culture is more straightforward than developing 3D culture systems, which has allowed scientists to obtain various 2D neuromuscular co-culture models [12–14]; but these models have relevant drawbacks. For instance, when using 2D-cultured myoblasts, differentiation, and subsequent spontaneous contractions of formed myotubes are known to cause detachment of cells from the substrate. To address this issue, it has been proposed to use a hydrogel overlay to ensure cell survival and support appropriate myotube contraction [11]. This 2D method has been largely used in our lab (S1 Fig) but lacked alignment of cells and myotubes adopted a random organization. Alternatively, surface modifications can improve attachment and alignment of individual myotubes [13], but do not recapitulate the formation of compacted bundles of myotubes similar to muscle myofibers.

When using 3D models, another advantageous feature is the ability to mimic the way that muscles are anchored to tendons, which connect the muscle to the bone. For this purpose, free-floating cell-laden hydrogel constructs have been developed, which can be anchored at both ends using different methods. One of the most popular methods is the use of flexible polydimethylsiloxane (PDMS) pillars around which the cells can attach [15–18]. This method has the advantage of allowing quantification of muscle contractile force, through observation of the flexible pillars’ deflection [19, 20] in response to muscle contraction. However, the microscale fabrication of these PDMS-based platforms is complex and time-consuming, often requiring use of cleanroom facilities for fabrication of molds by soft lithography. In addition, other types of anchoring methods without pillars, such as adding hydrogel anchors at the ends of a tubular mold [21, 22] suffer from a higher rate breakage or anchor detachment, which results in the loss of putatively valuable samples after long culture times. Finally, due to the large number of myoblasts needed to ensure proper myotube formation and the small chambers used for these purposes, cultures often display poor oxygen and nutrient diffusion in generated muscle fascicles, affecting cell survival.

As a simplified yet effective alternative, several publications have relied on the use of pieces of VELCRO^TM^ fabric as anchors for bioengineered muscle constructs [4, 5, 23, 24]. In this method, the loops on the VELCRO^TM^ successfully entrap the hydrogel-cell mixture, forming a robust anchoring site. Attaching the VELCRO^TM^ pieces can be achieved by simply gluing them to the culture support of choice. In particular, the simplest manufacturing method is the one published in [4], which directly glues VELCRO^TM^ pieces onto 6-well culture plates using PDMS glue. Nevertheless, in this method, the selected hydrogel composition (a mixture of Matrigel^TM^ and Collagen I) differs from the most commonly used hydrogel (Matrigel^TM^ and Fibrinogen) in other studies using myoblasts [5, 17, 24, 25], which was reported to show the best properties to support muscle differentiation and function in a comparative study on ECM composition [23]. In fact, in our experience, the Matrigel^TM^/Collagen I based gels showed an excessive degree of compaction over time, which resulted in excessive rigidity, low oxygen and nutrient diffusion, increased cell death, and breakage of muscle bundles.

Therefore, we were interested in optimizing a simple culture system combining VELCRO™ anchors and the Matrigel^TM^/Fibrinogen hydrogel composition. We noticed that, in anchor-based systems, this hydrogel is always used in combination with some type of mold coated with a hydrophobic Pluronic^®^ solution [5, 23] or bioprinted [17, 25] to achieve the desired shape. This is likely due to the lesser degree of compaction of this gel, which results in an inability to detach from the culture dish on its own, as compared to the Matrigel^TM^/Collagen-based gel, which readily detaches with no coating [4]. Thus, we modified the protocol to ensure proper fascicle formation and contraction with easy manipulation and contraction analysis. Indeed, here we use three different kinds of stimulation and two different ways to quantify muscle response in our modified platforms. The protocol has been adapted to allow for individual imaging of muscle bundles, by using cell culture 35 mm ∅ Petri dishes.

The most relevant indicator of functional maturation in muscle cells is their contractile ability. *In vivo*, muscle contraction is initiated by motor neurons, which innervate muscles at the end-plate. In the vicinity of this area, neuron terminals branch into synaptic boutons releasing the neurotransmitter acetylcholine (ACh), which binds to nicotinic acetylcholine receptors (AChRs) on the muscle membrane [26]. This event causes rapid depolarization of the membrane, resulting in a large post-synaptic excitatory potential [26]. This mechanism of contraction implies that *in vitro* stimulation of muscle constructs can be achieved either by chemical stimulation with acetylcholine or other depolarizing agents (e.g., potassium chloride), or simply by applying electrical pulses to depolarize the cell membrane.

Electrical stimulation is widely used in *in vitro* models to study contractile response [20, 23, 25], as well as for maturation and hypertrophy of muscle constructs [27–29]. In this protocol, we use a simple graphite electrode setup for applying electrical pulses to individual plates. The muscle constructs generated with this protocol reproducibly respond to electrical stimulation, showing synchronic contractions. However, despite the effectiveness of electrical stimulation, this stimulus differs from physiological conditions, and may cause excessive depolarization of muscle cells. For this reason, we have also performed chemical stimulation with acetylcholine [5, 15]. In the absence of motor neuron co-culture, we have induced clustering of AChRs by the addition of agrin to the differentiation medium [5, 30].

As an alternative stimulation method, we have included the option of using optogenetic stimulation in our muscle constructs by modifying cultured myotubes to express the genetically encoded light-activated ion channel Channelrhodopsin-2 (ChR2) [22]. As this method is based on the selective activation of a single ion channel, optical stimulation is a gentler method to depolarize cells, as opposed to a general electrical stimulus. In addition, using light is also non-invasive, which reduces the possibility of contamination derived from the introduction of external electrodes or chemical solutions [20].

After the application of these three types of stimulation, we illustrate two different methods for evaluating muscle response, which are contraction analysis in bright-field time-lapse videos, or calcium imaging with the fluorescent calcium indicator Fluo-4 AM. The first method is based on the acquisition of sequential images of contraction using bright-field, from which displacement and contraction speed data is extracted in relation to pixel movement with the MUSCLEMOTION plugin for ImageJ [31, 32]. Thus, this method is compatible with all three proposed stimulation methods. Alternatively, we have also included the option to perform calcium imaging. Calcium changes can be easily analyzed by using a fluorescent calcium indicator such as Fluo-4 AM [33], or a genetically encoded calcium indicator (GECI) such as GCaMP6 [34]. In this protocol, muscle bundles are loaded with Fluo-4 AM and imaged during stimulation with a fluorescence microscope and blue light filter. Then, videos are processed using ImageJ to extract normalized fluorescence data from a set of cell ROIs of each muscle bundle, and quantify fluorescence increases that occur due to stimulation. In all, with the combination of these methods, this step-by-step protocol can serve as a practical guide for studying muscular activity in a versatile manner, while using a straightforward and reliable setup.

## Materials and methods

The protocol described in this peer-reviewed article is published on protocols.io, updated February 11 2022, **dx.doi.org/10.17504/protocols.io.b4qiqvue** and is included for printing as S1 File with this article.

### Expected results

**Fig 1:**
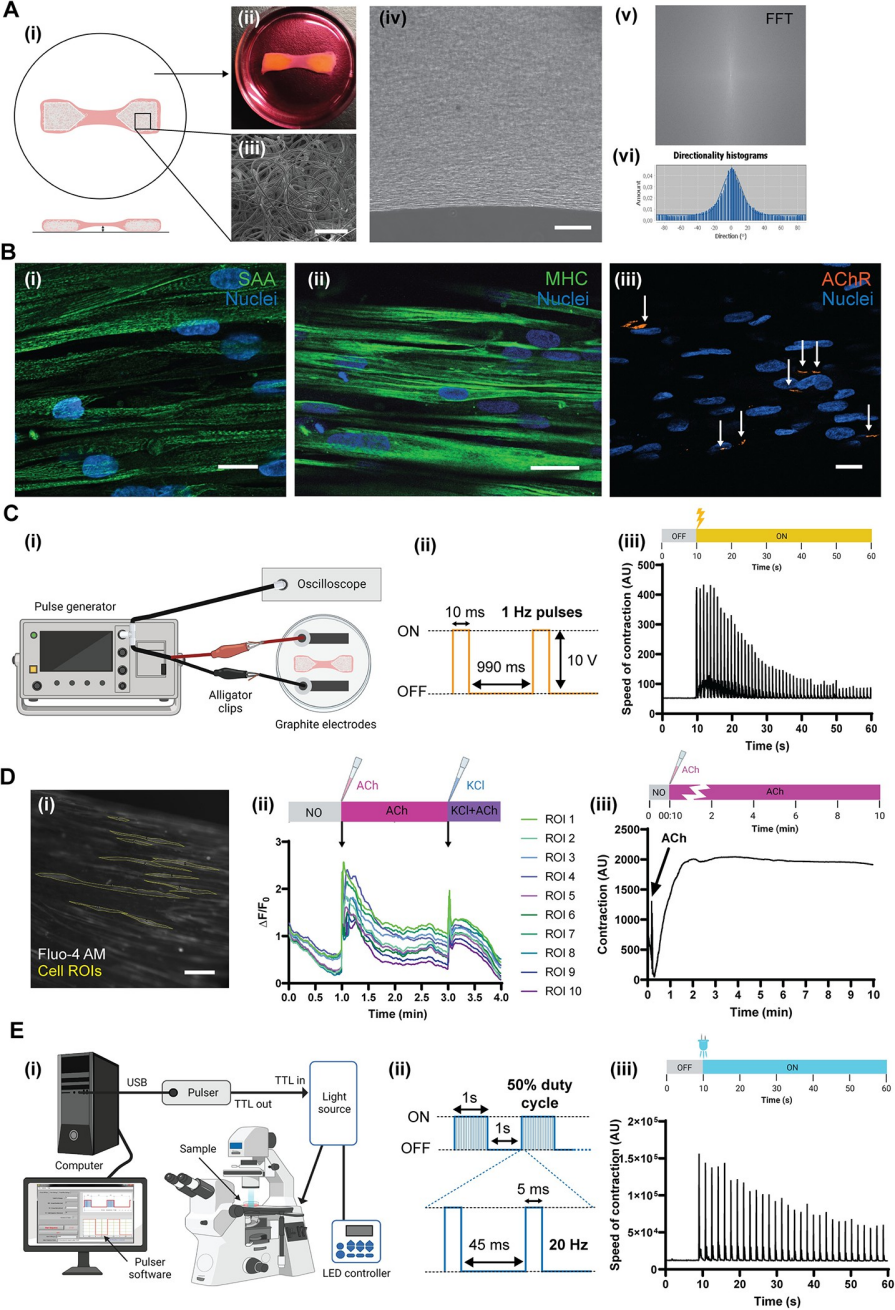
Expected results. **A)** Example of the generation of the muscle constructs. (i) Scheme illustrating the establishment of the muscle strip between the two VELCRO^TM^ pieces. Photograph of the construct shown in (ii). SEM analysis of the anchor microstructure can be seen in (iii). (iv) Low phase contrast photomicrograph illustrating the dense band of oriented myotubes. This orientation can be easily analyzed by using the ImageJ Directionality plugin (v and vi). **B)** Imaging of differentiation markers by immunostaining and confocal fluorescence microscopy: sarcomeric α-actinin (SAA) and Hoechst 33342 (i), MHC and Hoechst 33342 (ii) or α-BTX and Hoechst 33342 (iii). Note the generation of sarcomeric banding patterns in (i) and (ii), as well as end-plate formation (arrows in iii). **C)** Electrical stimulation. A scheme of the basic stimulation setup can be seen in (i). (iii) Example of the analysis of the myotubes contraction by using MUSCLEMOTION. In this analysis, the electrical stimulation was performed as indicated in (ii). **D)** Chemical stimulation and calcium imaging. Example of ROI selection with ImageJ in mytotubes incubated with Fluo4-AM (i). In this example, 10 ROIS are displayed, and the graph illustrating the calcium changes (indicated as ΔF/F_0_) after ACh and KCl treatment is shown in (ii). (iii) Example of contraction analysis of a classical ACh treatment in our device, analyzed with MUSCLEMOTION. **E)** Optical stimulation. (i) Scheme of the basic setup for optogenetic stimulation of our devices using an inverted microscope, Pulser device, and LED light source. (iii) Example of the contraction analysis using the light exposure protocol illustrated in (ii) after their analysis using ImageJ. Scale bars: **A)** (ii) 1 mm, (iii) 1 mm, (iv) 200 μm. **B)** (ii) 15 μm (ii) 15 μm, (iii) 15 μm. **D)** (i) 100 μm.

In the following paragraphs, we describe some of the results that can be obtained using our protocol, as illustrated in Fig 1.

### Validation of muscle bundle formation

After the culture of the myoblast cell line (see Materials and Methods), the first part of the validation process consists in verifying that there is an appropriate development of differentiated muscle bundles. In our protocol, we used human myoblasts (AB1079 cell line), which typically require 2 weeks in differentiation medium to mature. Before this occurs, it is critical that the muscle bundles detach from the plate due to the addition of a Pluronic^®^ coating (Fig 1A). This typically occurs between days 1 and 3 after seeding, and can be seen by simply looking at the plates by eye. During the differentiation of muscle bundles, bright-field images can be taken in order to evaluate alignment of the cells within the construct. Myoblasts should show a progressively aligned morphology, beginning with the outermost region and progressing over time throughout the construct. In order to quantitatively evaluate alignment, images may be processed using the Directionality plugin for ImageJ (Fig 1A).

### Muscle differentiation

After differentiation, confocal imaging can be performed in order to evaluate expression of differentiation markers. We recommend staining of three key markers: sarcomeric α-actinin (SAA), myosin heavy chain (MHC) and acetylcholine receptor (AChR) clusters (Fig 1B). For SAA and MHC, it is necessary to confirm that cells possess a sarcomeric banding pattern (Fig 1B), characteristic of mature and contractile cells. SAA staining is more specific for late-stage differentiation than MHC staining. Regarding AChR receptor clusters, distinct accumulations of fluorescent α-bungarotoxin (α-BTX) should be observed on the cell membranes (Fig 1B). Multinucleated cells will also be seen in confocal images by nuclear staining.

### Electrical stimulation

In Fig 1C, we illustrate the classical configuration used for electrical stimulation, consisting of a pulse generator connected to a digital oscilloscope and to the electrodes used for the cells (see additional details for setup in S2 Fig). In addition, a proposed stimulation protocol is illustrated, using pulses of 1 Hz and 10 V. The analysis of the generated “pacemaker” contraction after this stimulation can be observed in Fig 1C. Note that after maintained stimulation, the degree of contraction of generated myotubes decreases, as easily observed by using MUSCLEMOTION software.

### Chemical stimulation and calcium analysis

As indicated above, the response of myotubes to ACh can be followed by using Fluo4-AM or by using MUSCLEMOTION. Examples of selected ROIs of Fluo4-AM loaded myotubes can be seen in Fig 1D. After determination of the changes in fluorescence over time, the values of ΔF/F_0_ can be easily calculated (see Materials and Methods) and displayed for each ROI in a graph (Fig 1D). In the example, a double pharmacological treatment is performed by sequentially adding ACh at 1 min of the time-lapse, and KCl after 3 min. It is common to see a lower response to KCl after the cells have been depolarized with ACh, as this treatment is not removed from the medium. Regardless, the addition of KCl serves as a positive control of depolarization of the muscle cells. It is important to note that there is an immediate peak of fluorescence at the moment when the chemical solution is added, which is then followed by a more gradual increase after a few seconds. This second peak corresponds to the calcium release in response to the depolarizing agent. Additionally, in the beginning of the recording, there is a progressive decrease in fluorescence due to an (expected) initial quenching of Fluo-4 fluorescence. In parallel to calcium analysis, contraction can be analyzed by MUSCLEMOTION (Fig 1D). In this case, cells show a gradual contraction response which results in maintained tetanization of the bundle while the ACh is present in the medium.

### Optical stimulation

Our setup uses a CoolLED device (see reference in the Materials and Methods section) directly connected to an inverted fluorescence microscope, as this system allows for the application of digital computer signals (TTL pulses). A scheme of our system is illustrated in Fig 1E. The setup provides an excellent platform for the study of muscle contraction as was previously shown in our studies [22]. As an example, a stimulus of 20 Hz with a 50% duty cycle is also illustrated (Fig 1E). The Pulser software can monitor the generation of the pulses in real time, which allows the user to follow the behavior of the cultured myotubes in response to the stimuli. An example of these experiments can be seen in Fig 1E. In the graph, synchronic contraction can be observed after each light pulse. Similar to the electrical stimulation graph, a progressive decrease in contraction can also be noted.

## Supporting information

S1 File Step-by-step protocol, also available on protocols.io. (PDF)

S1 Fig 2D differentiation protocol with hydrogel overlay. Phase contrast photomicrographs of three myoblast cell lines (LHCN-M2, AB1079, and 8220) differentiated for 14–17 days on gelatin-coated coverslips. To avoid myotube detachment, cells were covered with an overlay of Matrigel^TM^ Growth Factor Reduced (GFR) Basement Membrane Matrix (1:3 dilution in DMEM). Note the lack of clear alignment between myotubes. Scale bars: A = 100 μm pertains to B-C.
(TIF)

S2 Fig Details of sample setup for electrical stimulation. **A)** Overall view of stimulation equipment and setup. The pulse generator is connected to the electrodes via a connection to a pair of alligator clips, and also to an oscilloscope (use a T connector to achieve this dual connection). **B)** Placement and fastening of the sample to the microscope stage. Tape is placed on the plate to fasten the lid to the microscope holder, and also on the alligator clips to avoid movement during focusing. The electrodes must remain parallel to the construct. **C)** Close-up of the stimulation device placed on a muscle construct. See how the wires are wrapped around the graphite rods in **D)**. Wires are sealed to the lid and kept in place with Loctite® Super Glue.
(TIF)

S1 Dataset Electrical stimulation contraction data obtained using MUSCLEMOTION software. Used to generate contraction graph in Fig 1C.
(XLSX)

S2 Dataset Fluorescence information extracted from Fluo-4 AM calcium transient recording with ImageJ. Used to generate calcium transient graph in Fig 1D.
(XLSX)

S3 Dataset ACh stimulation contraction data obtained using MUSCLEMOTION software. Used to generate contraction graph in Fig 1D.
(XLSX)

S4 Dataset Optical stimulation contraction data obtained using MUSCLEMOTION software. Used to generate contraction graph in Fig 1E.
(XLSX)
