# Supplementary material for: Development of a simple and versatile in vitro method for production, stimulation, and analysis of bioengineered muscle
Source: PLoS One. 2022 Aug 11;17(8):e0272610. doi: 10.1371/journal.pone.0272610 (PMC9371355; doi:10.1371/journal.pone.0272610)
Supplement: S1 File — (PDF) [file pone.0272610.s001.pdf]

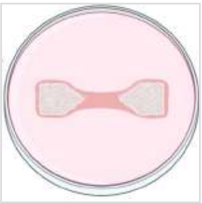

Development of a simple and versatile *in vitro* method for production, stimulation, and analysis of bioengineered muscle

Karen Wells-Cembrano[1,2,3,4], Júlia Sala[1,2,3,4], Jose Antonio del Rio[1,2,3,4]

[1]Molecular and Cellular Neurobiotechnology, Institute for Bioengineering of Catalonia (IBEC), Scienti fic Park of Barcelona, The Barcelona Institute for Science and Technology (BIST), Barcelona, Spain.;

[2]Department of Cell Biology, Physiology and Immunology, University of Barcelona, Barcelona, Spain.;

[3]Network Centre of Biomedical Research of Neurodegenerative Diseases (CIBERNED), Institute of H ealth Carlos III, Ministry of Economy and Competitiveness, Spain.;

[4]Institute of Neuroscience, University of Barcelona. Barcelona, Spain.

dx.doi.org/10.17504/protocols.io.81wgb6ojolpk/v1

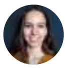

Karen Wells-Cembrano

ABSTRACT

In recent years, 3D *in vitro* modeling of human skeletal muscle has emerged as a subject of increasing interest, due to its applicability in basic studies or screening platforms. These models strive to recapitulate key features of muscle architecture and function, such as cell alignment, maturation, and contractility in response to stimuli. To this end, it is required to culture cells in biomimetic hydrogels suspended between two anchors. Currently available protocols are often complex to produce, have a high rate of breakage, or are not adapted to imaging and stimulation. Therefore, we sought to develop a simplified and reliable protocol, which still enabled versatility in the study of muscle function. In our method, we have used human immortalized myoblasts cultured in a hydrogel composed of Matrigel^{TM} and fibrinogen, to create muscle strips suspended between two VELCRO^{TM} anchors. The resulting muscle constructs show a differentiated phenotype and contractile activity in response to electrical, chemical and optical stimulation. This activity is analyzed by two alternative methods, namely contraction analysis and calcium analysis with Fluo-4 AM. In all, our protocol provides an optimized version of previously published methods, enabling individual imaging of muscle bundles and straightforward analysis of muscle response with standard image analysis software. Although limited by the lack of co-culture with motor neurons, this system provides a start-to-finish guide on how to produce, validate, stimulate, and analyze bioengineered muscle. This ensures that the system can be quickly established by researchers with varying degrees of expertise, while maintaining reliability and similarity to native muscle.

DOI

dx.doi.org/10.17504/protocols.io.81wgb6ojolpk/v1

PROTOCOL CITATION

Karen Wells-Cembrano, Júlia Sala, Jose Antonio del Rio . Development of a simple and versatile in vitro method for production, stimulation, and analysis of bioengineered muscle. **protocols.io**
https://dx.doi.org/10.17504/protocols.io.81wgb6ojolpk/v1

KEYWORDS

Bioengineered muscle, skeletal muscle, in vitro model, electrical stimulation, optogenetics, acetylcholine, muscle stimulation, hydrogels, biomaterials, differentiation, Matrigel, Fibrinogen, Velcro, 3D model, ChR2, Fluo-4 AM, ImageJ

LICENSE

This is an open access protocol distributed under the terms of the Creative Commons Attribution License, which permits unrestricted use, distribution, and reproduction in any medium, provided the original author and source are credited

IMAGE ATTRIBUTION

Figures created with BioRender.com

CREATED

Jun 29, 2022

LAST MODIFIED

Jun 30, 2022

PROTOCOL INTEGER ID

65633

White Velcro fabric (no adhesive backing). White color ensures that no dyes can leach into the culture.

35 mm ∅ Petri dishes (Cell culture treated)

Sylgard 184 **Dow Corning** Step 2

Skeletal Muscle Cell Growth Medium **PromoCell Catalog #C-23160** Step 6

Thrombin from bovine plasma **Sigma Aldrich Catalog #T4648** Step 8

HBSS, no calcium, no magnesium, no phenol red **Thermo Fisher Catalog #14175046** Step 8

Matrigel Growth Factor Reduced (GFR) Basement Membrane Matrix, LDEV-free **Corning Catalog #356230** Step 9

Fibrinogen from bovine plasma Type I-S 65-85% protein (≥75% of protein is clottable) **Sigma Aldrich Catalog #F8630** Step 10

Pluronic® F-127 **Sigma Aldrich Catalog #P2443** Step 12

DMEM **Thermo Fisher Scientific Catalog #41966** Step 15

M199 medium **Fisher Scientific Catalog #MT90050PB** Step 15

L-Glutamine (200mM) **Thermo Fisher Scientific Catalog #25030024** Step 15

Insulin from bovine pancreas **Sigma Aldrich Catalog #I6634-50MG** Step 15

Fetal Bovine Serum qualified heat inactivated Brazil **Gibco - Thermo Fisher Catalog #10500064** Step 15

Recombinant Rat Agrin Protein **R&D Systems Catalog #550-AG-100** Step 15

Penicillin-Streptomycin **Sigma Aldrich Catalog #P4333** Step 15

Amphotericin B **Thermo Fisher Scientific Catalog #15290026** Step 15

6-Aminocaproic acid **Sigma Aldrich Catalog #A7824-100G** Step 15

Goat Serum, New Zealand origin **Thermo Fisher Catalog #16210064** In 2 steps

Monoclonal Anti-α-Actinin (Sarcomeric) antibody produced in mouse **Sigma Aldrich Catalog #A7811** Step 18.4

Anti-Myosin (Skeletal Fast) antibody Mouse monoclonal **Sigma Aldrich Catalog #M1570** Step 18.4

Goat anti-Mouse IgG (H L) Highly Cross-Adsorbed Secondary Antibody Alexa Fluor 488 **Thermo Fisher Scientific Catalog #A-11029** Step 18.4

α-Bungarotoxin, Alexa Fluor™ 555 conjugate **Thermo Fisher Catalog #B35451** Step 18.4

Mowiol® 4-88 **Sigma Aldrich Catalog #81381-50G** Step 18.5

Acetylcholine chloride **Sigma Aldrich Catalog #A6625** Step 21

Potassium chloride (KCl)

Fluo-4, AM, cell permeant **Thermo Fisher Catalog #F14217** Step 36

Dimethyl sulfoxide (DMSO) **Sigma Aldrich Catalog #D2650** Step 36

SAFETY WARNINGS

Follow BSL-2 guidelines for handling **human cell lines**.

Paraformaldehyde is a toxic chemical, handle in a fume hood and wear appropriate PPE (gloves, lab coat).

α-Bungarotoxin is a potent neurotoxin. At the used concentrations and in a standard manipulation setting, it is not considered a hazardous solution, but should be handled with care (gloves, avoid spills/skin exposure/ingestion).

BEFORE STARTING

Protocol overview:

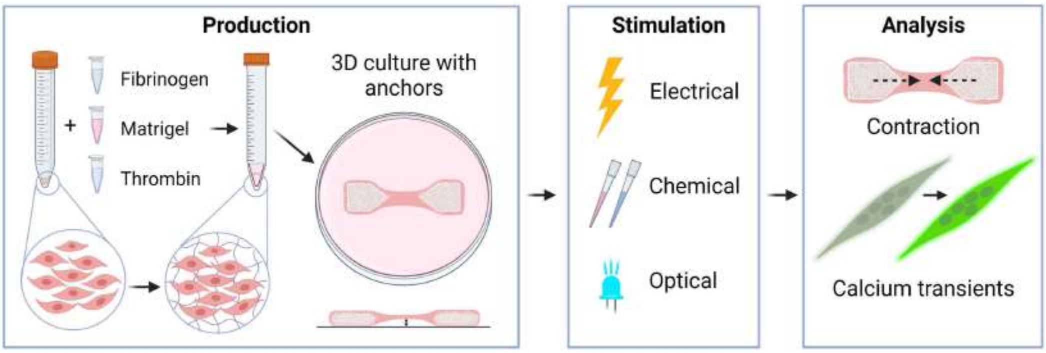

General workflow of the protocol

Making muscle culture devices

1h 30m

1 **Prepare VELCRO® pieces.** Only use white velcro without adhesive backing. Using only the "loop" (soft) part of the VELCRO®, cut arrow-shaped velcro pieces according to directions in **Figure 1**. Use a pencil (not a marker) to divide the VELCRO® into 12 x 4 mm strips, as shown in **Figure 1**. Then, cut these strips in half (**Figure 1A**), which can be easily done by folding the strip and cutting along the fold. Next, cut the arrowheads as shown in **Figure 1B**.

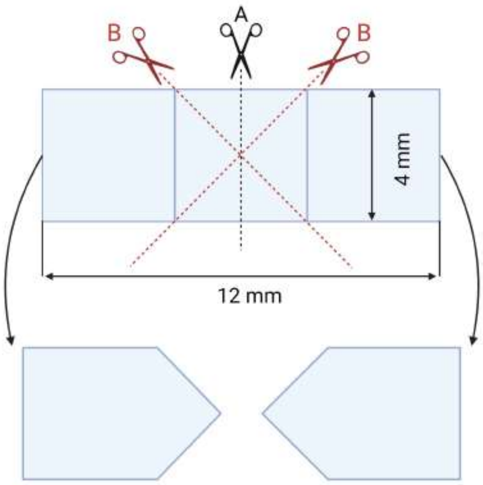

Figure 1. How to cut VELCRO® pieces.

2 **Prepare PDMS glue** by mixing Sylgard 184 **Dow Corning** components at a 10:1 ratio. 1h

Weigh 10 parts of elastomer and 1 part of curing agent in a plastic cup. Mix the components thoroughly with a glass rod, until the mixture is full of small bubbles and opaque in appearance. Then, leave the mixture in a vacuum dessicator for **01:00:00** to completely remove bubbles.

PDMS glue can be prepared in bulk, and stored at **-20 °C** until the next preparation of muscle culture plates. When needed, it can be brought back to room temperature or briefly heated in a **37 °C** water bath to reduce viscosity. This is convenient for reducing the total preparation time in subsequent batches of plates.

3 **Mark** a line of 1.2 cm in the center of each 35 mm ∅ Petri dish. In order to obtain consistently centered markings, it is recommended to make a small stencil out of paper or plastic (**Figure 2**).

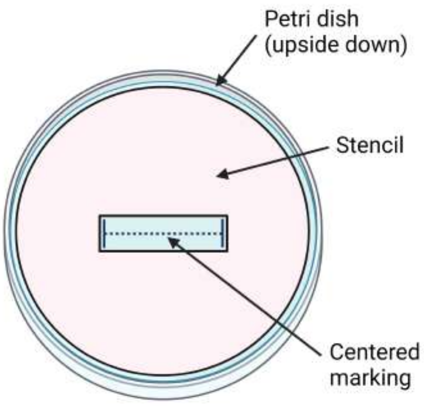

Figure 2. Stencil to make centered markings on Petri dishes.

If desired, the permanent marker lines can be erased with 70% ethanol after preparation to avoid capturing them in microscopy images.

4 **Attach VELCRO® anchors** to the Petri dishes, using the marked line as a guide. Use a small spatula to dab glue onto the underside of the VELCRO® pieces, and then place them on the lines using tweezers, as shown in **Figure 3**. Make sure that the tips of the anchors are aligned, and the ends of the markings are aligned to the start of the arrowhead region of the anchors.

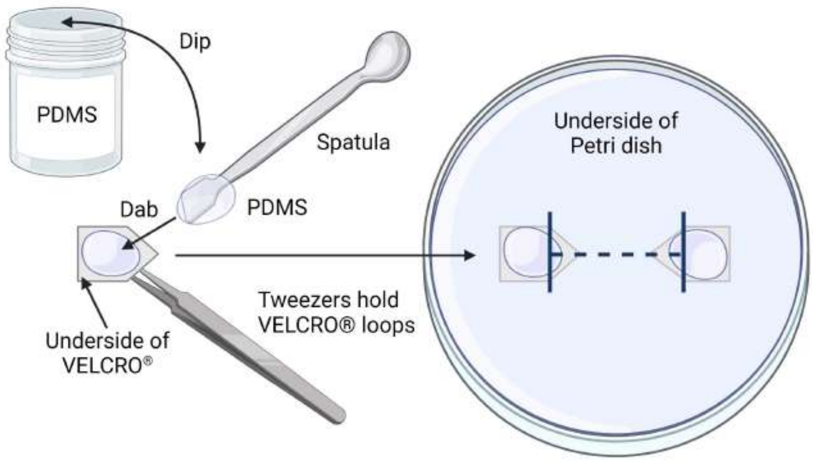

Figure 3. Attachment of VELCRO® anchors to Petri dishes using PDMS glue.

5 Once the VELCRO® pieces are attached, leave the plates at Room temperature Overnight to **cure the PDMS glue**. Then, store them until further use.

Preparation of cells and materials 20m

6 ⚠ 1h

In this section, all steps are performed inside a biological safety cabinet (vertical flow / BSL-2 preferrable).

Defrost myoblast cell line in a T75 flask and culture cells in their recommended maintenance medium. Human myoblasts must be passaged at a maximum of 60-70% confluence to avoid differentiation induced by cell-cell contacts.

For maintenance of human myoblasts, we use

Skeletal Muscle Cell Growth Medium **PromoCell Catalog #C-23160** . Culture flasks are coated with a solution of 0.15% gelatin for 01:00:00 before use.

7 **Amplify cells** to the needed amount. Take into account that around **3·10^6 cells are needed for each muscle bundle**. If many replicates are to be seeded, a large number of flasks and high volume of cell culture medium may be necessary depending on the yield for each cell line. In the case of human myoblasts, cells are sometimes larger than other common cell lines, which can make it more difficult to obtain high yields of cells while keeping confluence under 70%.

Make sure to become familiarized with the culture time and passage dilutions required for your specific muscle cell line, as well as usual yields of cells per T75 or T175 flask at ~70% confluence. This is necessary to ensure that the required number of cells will be available at the day of the experiment.
For example, in our case, we obtain approximately 6·10^6 cells in one T175 flask. Therefore, we make sure to have at least one T175 at 70% confluence for every two replicates on the day of the experiment. We perform passages at a 1:5-1:10 dilution.

8 **Prepare a thrombin stock solution** according to manufacturer's instructions, using

Thrombin from bovine plasma **Sigma Aldrich Catalog #T4648** . Prepare a stock of 100U/ml, to be frozen in individual aliquots. Then, on the day of seeding, dilute these aliquots 1:5 in HBSS (

HBSS, no calcium, no magnesium, no phenol red **Thermo Fisher Catalog #14175046**) to obtain a 20U/ml solution.

9 The day before the seeding, defrost the appropriate amount of 2h

Matrigel Growth Factor Reduced (GFR) Basement Membrane Matrix, LDEV-free **Corning Catalog #356230**

. For our experiments, a concentration of 7.2-8 mg/ml has been used with similar results. Leave aliquots on ice inside the fridge **Overnight** . It is recommended to start defrosting in the afternoon, and to change the ice the next morning.

10 The day of the seeding, prepare a **stock solution of fibrinogen** at [M] **8 mg/mL** .

Fibrinogen from bovine plasma Type I-S 65-85% protein (≥75% of protein is clottable) **Sigma Aldrich Catalog #F8630**

.

First, calculate the necessary amount of fibrinogen solution needed for your experiment, taking into account that **150 µL** are needed for each muscle bundle. Prepare 1.5 times the necessary volume.

Layer fibrinogen over warm (sterile) phosphate-buffered saline (PBS), leave it in the 37 °C water bath to fully dissolve (DO NOT VORTEX), and filter through a 0.22 µm filter. Keep the solution at 4 °C until use.

It is best to prepare the fibrinogen solution fresh, as it only keeps for <7 days at 4 °C .

11 On the day of seeding, **sterilize the necessary amount of Petri dishes** according to the following steps.

TIP: To the desired number of replicates, add at least two more Petri dishes for sterilization, which can be used as replacements in case one of the VELCRO® pieces becomes detached during the sterilization process (this occurs in less than 10% of plates).

11.1 Place the Petri dishes on larger (150 mm Ø or similar) Petri dishes, for ease of manipulation (**Figure 4**). Add 70% ethanol to each dish, up to the brim, and leave for 00:15:00 . 15m

11.2 Remove ethanol and wash 3 times with sterile PBS. Without removing the 3rd wash, leave the plates under UV light for 15 minutes (lid open and facing upwards as in **Figure 4**).

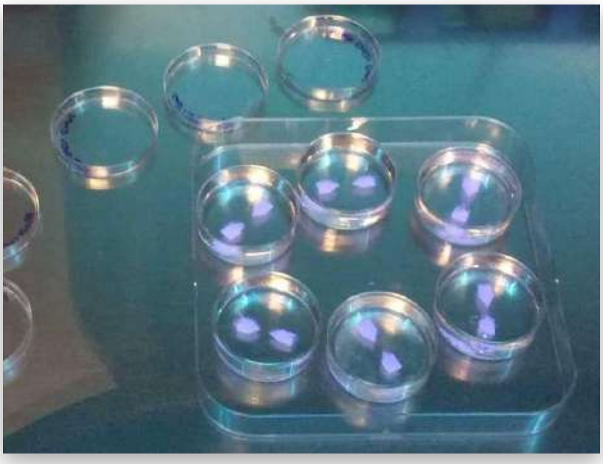

Figure 4. Sterilization of plates with PBS under UV light.

11.3 After UV sterilization, turn the hood back on, and remove the PBS by aspiration with a glass Pasteur pipette and vacuum. Make sure to aspirate all of the liquid from the VELCRO® anchors, which are quite absorbent. Then, leave the plates in the incubator for approximately 01:00:00 to dry the plates completely. 1h

12 After the plates have been dried in the incubator, perform coating with a sterile solution of 15m

[M]**0.2 Mass / % volume** Pluronic® F-127 **Sigma Aldrich Catalog #P2443** in Milli-Q® water.

This hydrophobic coating will facilitate detachment of the muscle bundles from the Petri dish. In order to coat only the middle region where the muscle bundle will be placed, and avoid soaking the velcro with a hydrophobic material, the Pluronic® solution is applied with a sterile fine paintbrush. Sterilize the paintbrush by immersion in 70% ethanol for **00:15:00** , and dry by aspiration with vacuum. Ideally, choose a paintbrush with short and square bristles.

12.1 Apply Pluronic® solution according to **Figure 5**. Ensure that the region is completely covered, by dipping the paintbrush into the solution and reapplying several times if needed.

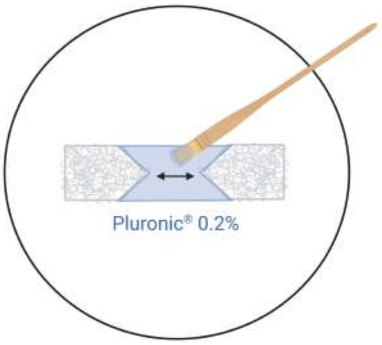

Figure 5. Region for application of Pluronic® coating.

12.2 Once all plates are coated, leave the dishes in the incubator (**37 °C** and 1h

[M]**5 % (v/v)** CO_2) for **01:00:00** . Importantly, during this time, the coating solution will completely evaporate, leaving only the solute on the surface. After this, it is not necessary to aspirate or wash the coating solution; the hydrogel is directly seeded onto the plates. In this way, the plates will remain completely dry at the time of seeding, which will enable correct spreading of the hydrogel as shown in **Step 14.5**.

Seeding and differentiation of muscle bundles

13 **Trypsinize cells** for seeding according to standard procedures. A large volume of cell suspension may be obtained depending on the number of devices to be seeded, so for comfort, it can be collected in one of the used flasks before counting. From there, take an aliquot of the cell suspension and count the cells. 5m

Then, **calculate the volume needed for obtaining $3 \cdot 10^6$ cells**, and place this volume into individual Falcon tubes. Each Falcon tube will contain enough cells for one muscle bundle.

Centrifuge cells at 800 rpm for **00:05:00** .

14 After centrifuging cells, place the Falcon tubes in the hood and use them sequentially to make muscle bundles. The cells will be resuspended in a hydrogel containing the components listed in **Figure 6**. All components must be kept on ice before and during the seeding procedure.

Component	Stock solution	Final concentration	Volume (µl)	% of final volume
Fibrinogen	8 mg/ml	4 mg/ml	150	50%
Matrigel GFR	7.2-8 mg/ml	3.6-4 mg/ml	120	40%
Thrombin	20 U/ml	2 U/ml	30	10%

Figure 6. Hydrogel stock solutions and final composition for one muscle construct.

14.1 First, aspirate the supernatant and resuspend the cell pellet in the indicated amount of fibrinogen (**150 µL**). Keep the Falcon tube on ice hereafter.

14.2 Then, add the indicated amount of Matrigel® (**120 µL**) and homogenize the mixture gently by pipetting.

14.3 After this step, prepare a P1000 pipette with a pipette tip and set it to **300 µL** . Set it aside until needed for seeding.

14.4 Add **30 µL** of thrombin solution to the tube, and then swiftly homogenize the contents of the tube using the P1000 pipette (4-5 times, gently). Aspirate all of the volume and take it to one of the Petri dishes.

14.5 ⚠

For **seeding the hydrogel-cell mixture**, quickly but carefully follow the directions in **Figure 7**. It is important to follow this order for distributing the liquid onto the VELCRO® and forming the correct shape.

1. First, add part of the solution to both VELCRO® anchors, and make sure to push the liquid into the loops by repeatedly pressing with the pipette tip in all directions. Due to the viscosity of the solution, it will not enter into the loops unless pressed into them, and this is important for forming a continuous structure with the middle portion.
2. Then, add most of the remaining solution to the middle part, and connect the two VELCRO® anchors to each other.
3. Finally, distribute the hydrogel around the anchors to ensure that all sides are covered.

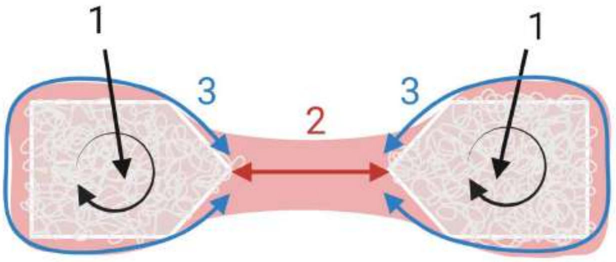

1. **Figure 7.** Procedure for correctly distributing the hydrogel solution onto the VELCRO® anchors.

After seeding, the resulting structure will look flat and wide (**Figure 8**), as the hydrogel is still attached to the plate. Nevertheless, the shape will improve upon differentiation and compaction of the muscle bundles.

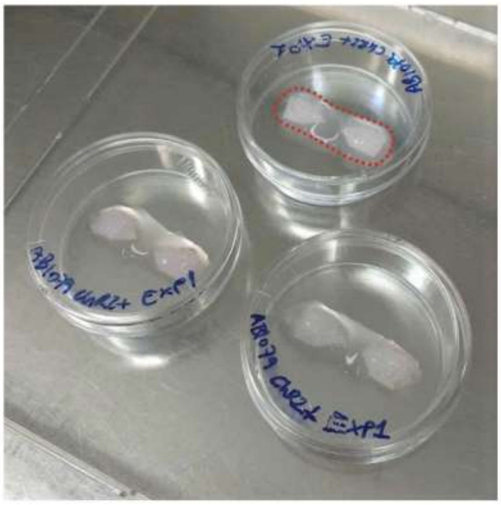

Figure 8. Appearance of muscle bundles after seeding. Dashed red line indicates example outline.

15 **After two days in maintenance medium, switch the cells to differentiation medium**. In **Figure 9**, the composition of differentiation medium for AB1079 cells is shown. This medium is prepared using a 3:1 mixture of DMEM and M199, respectively. Agrin is used for mimicking neural signaling for AChR clustering. 6-Aminocaproic acid (6-ACA) is used as an antifibrinolytic agent, which aids in the long-term maintenance of the structure.

Component	Final concentration	For 10 ml
DMEM 41966	3 parts	7,1 ml
M199	1 part	2,4 ml
Glutamine	1%	100 µl
Insulin (Stock 10 mg/ml)	10 µg/ul	10 µl
FBS	2%	200 µl
Agrin (Stock 500 µg/ml)	100 ng/ml	2 µl
P/S	0,9%	90 µl
Fungizone	0,1%	10 µl
6-ACA (Stock 100 mg/ml)	1 mg/ml	100 µl

Figure 9. Differentiation medium for AB1079 myoblasts.

References of used materials:

- DMEM 41966: DMEM **Thermo Fisher Scientific Catalog #41966**
- M199: M199 medium **Fisher Scientific Catalog #MT90050PB**
- Glutamine: L-Glutamine (200mM) **Thermo Fisher Scientific Catalog #25030024**
- Insulin: Insulin from bovine pancreas **Sigma Aldrich Catalog #I6634-50MG**
- FBS: Fetal Bovine Serum qualified heat inactivated Brazil **Gibco - Thermo Fisher Catalog #10500064**
- Agrin: Recombinant Rat Agrin Protein **R&D Systems Catalog #550-AG-100**
- P/S: Penicillin-Streptomycin **Sigma Aldrich Catalog #P4333**
- Fungizone: Amphotericin B **Thermo Fisher Scientific Catalog #15290026**
- 6-ACA: 6-Aminocaproic acid **Sigma Aldrich Catalog #A7824-100G**

Change half of the medium every 2 days (or right before and after a weekend), over the course of 14 days. **336:00:00** .

To prevent evaporation, keep muscle construct dishes inside a larger Petri dish (with lid) containing a small dish filled with sterile water.

Validation of muscle differentiation

Check that the muscle bundles detach from the plate within the first 1-3 days of culture. When seen from the side, the muscle bundles should be slightly lifted from the Petri dish as shown in **Figure 10**.

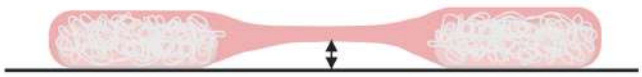

Figure 10. Detachment of muscle bundles from the Petri dish.

17 During differentiation, take brightfield images of the muscle bundles at 4X and 10X magnification. With these images, perform a directionality analysis to show **alignment of cells.** This can be done in ImageJ with the Directionality plugin, which will provide a Fast Fourier Transform (FFT) of the image, and a directionality histogram (**Figure 11**).

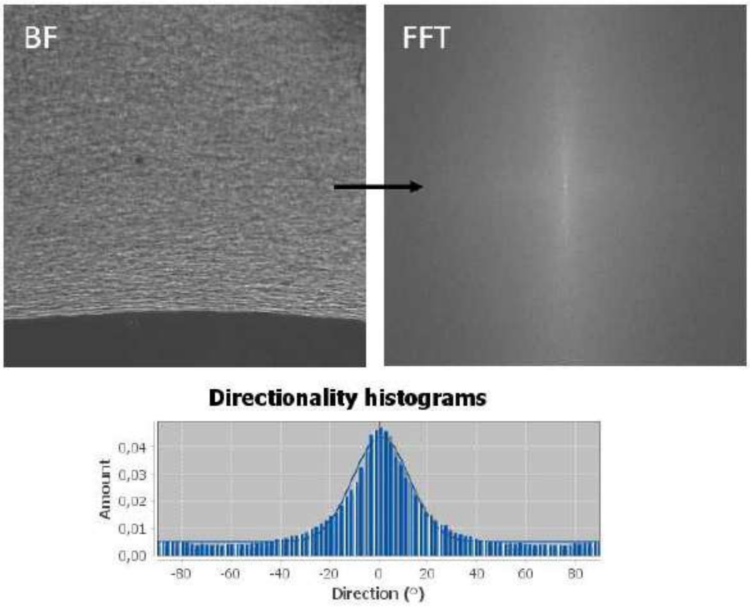

Figure 11. Directionality analysis of a brightfield image corresponding to cells differentiated for 14 days.

18 In order to confirm that differentiation has taken place, **perform immunostaining** of three key markers: Sarcomeric α-actinin (SAA), Myosin Heavy Chain (MHC) and acetylcholine receptors (AChR). The first two markers are needed to confirm the formation of sarcomeres (banding pattern), with SAA being more specific for late-stage differentiation. The latter is relevant if it is desired to perform chemical stimulation with acetylcholine. After staining, perform confocal imaging of the samples. It is recommended to view all of these markers at 40X magnification.

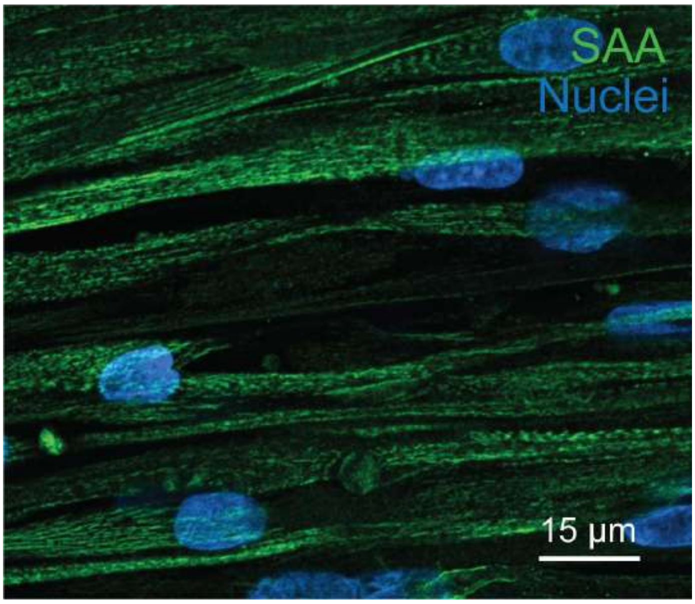

Figure 12. Confocal slice of SAA staining (green) at 40X magnification. Nuclei (blue) stained with Hoechst 33342.

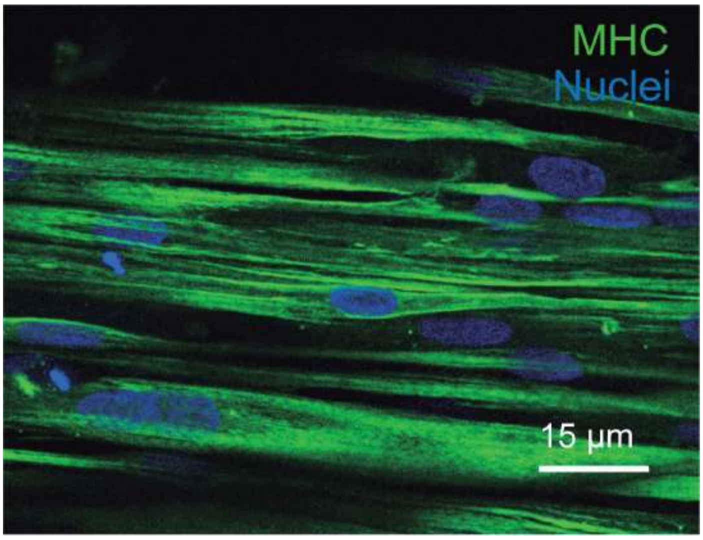

Figure 13. Confocal slice of MHC staining (green) at 40X magnification. Nuclei (blue) stained with Hoechst 33342.

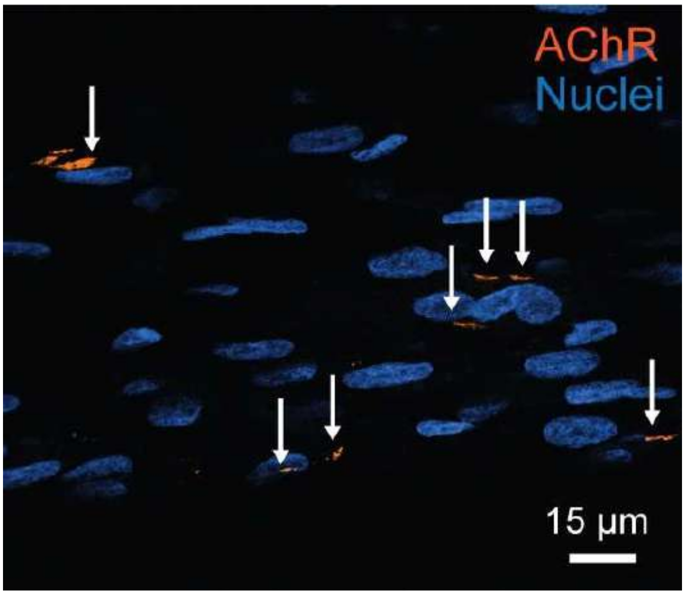

Figure 14. Confocal slice of AChR cluster staining (orange) with BTX-Alexa Fluor 555 at 40X magnification. Nuclei (blue) stained with Hoechst 33342 (1:200).

18.1 1h 40m

Fix the cells with phosphate-buffered paraformaldehyde (PFA) [M] **4 Mass / % volume** at **Room temperature** in a fume hood. First, add one volume of 4% PFA directly to the cell culture medium, to obtain a final concentration of 2% PFA. Incubate for **00:10:00** , aspirate, and fix for **00:30:00** to **01:00:00** in 4% PFA. After fixation, wash thoroughly with PBS (3 times) before taking the plates out of the fume hood.

> Do not fix cells on ice to avoid depolymerization of Matrigel®.

18.2 6h

Block the putative non-specific binding of the antibodies for **06:00:00** at **Room temperature** , in a blocking buffer of PBS - Gelatin [M] **1 Mass / % volume** with [M] **0.5 % (v/v)** Triton X-100 and [M] **10 % (v/v)** Goat Serum, New Zealand origin **Thermo Fisher Catalog #16210064** . Incubate the blocking solution with gentle shaking.

18.3

In order to reduce the necessary amount of antibody solutions and enable mounting, **cut the muscle constructs** using a scalpel as shown in **Figure 15.** Pick up the cut-out middle portions using a fine paintbrush, and place them in

the well of a 24-well plate. Note that the plate and well must contain PBS, in order to facilitate detachment of the hydrogel from the paintbrush.

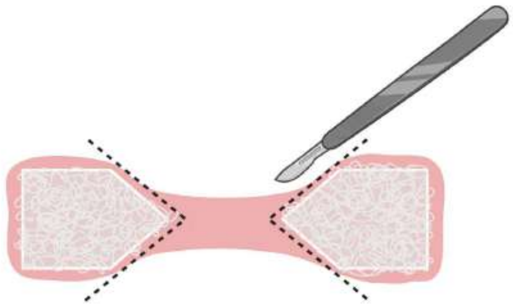

Figure 15. How to cut muscle constructs for staining and mounting.

18.4 **Perform antibody staining.** Prepare antibody or staining solutions in PBS - Gelatin [M]1 Mass / % volume with [M]0.5 % (v/v) Triton X-100 and [M]5 % (v/v) Goat Serum, New Zealand origin **Thermo Fisher Catalog #16210064** . All incubations and washes are performed with gentle shaking. Perform washes (3-4 washes of 00:10:00) between stainings, with PBS containing [M]0.5 % (v/v) Triton X-100.

18h 20m

Antibodies:

- **SAA:**
  Monoclonal Anti-α-Actinin (Sarcomeric) antibody produced in mouse **Sigma Aldrich Catalog #A7811**
  1:200 dilution. Incubate for **3** **Overnight** at **4 °C** , and follow up with Goat anti-Mouse IgG (H L) Highly Cross-Adsorbed Secondary Antibody Alexa Fluor 488 **Thermo Fisher Scientific Catalog #A-11029**
  , incubated for **06:00:00** at **Room temperature** to **Overnight** at **4 °C** .
- **MHC**:
  Anti-Myosin (Skeletal Fast) antibody Mouse monoclonal **Sigma Aldrich Catalog #M1570**
  1:400 dilution. Incubate for **3** **Overnight** at **4 °C** , and follow up with Goat anti-Mouse IgG (H L) Highly Cross-Adsorbed Secondary Antibody Alexa Fluor 488 **Thermo Fisher Scientific Catalog #A-11029**
  , incubated for **06:00:00** at **Room temperature** to **Overnight** at **4 °C** .
- **AChR** :
  α-Bungarotoxin, Alexa Fluor™ 555 conjugate **Thermo Fisher Catalog #B35451**

1:500 dilution. Incubate for **06:00:00** at **Room temperature** to **Overnight** at **4 °C** .

Finally, wash the samples 3 times for **00:10:00** with PBS containing [M]**0.5 % (v/v)** Triton-X100, and finally 3 times with PBS.

18.5 In order to **mount the samples** for confocal imaging, it is necessary to use coverslips with small drops of nail polish at the corners (prepare in advance and let them dry). The height of the dried nail polish will create a gap for the muscle bundle to be mounted without risk of excessive deformation (**Figure 16**).

Transfer the muscle bundles onto a glass slide using a fine paintbrush, and cover them with Mowiol® 4-88 **Sigma Aldrich Catalog #81381-50G** mounting medium. Then, place the modified coverslip on top of the constructs (without applying pressure) and let the mounting medium harden for 1-2 days at **4 °C** .

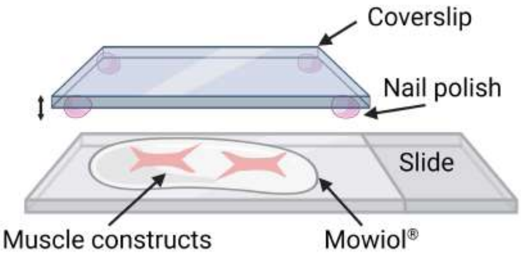

Figure 16. Mounting of muscle constructs using modified coverslips.

Electrical pulse stimulation

19 After 14 days in differentiation medium, cells can be stimulated electrically to prove that they have the ability to contract synchronically in response to electrical pulses.
Contractions may be imaged in brightfield and processed using the MUSCLEMOTION plugin for ImageJ, or alternatively, calcium imaging can be performed using Fluo-4 AM and subsequent $\Delta F/F_0$ analysis.

19.1 Before stimulation, it is necessary to build a simple **stimulation device**, which consists of two graphite electrodes attached to the lid of a 35 mm ∅ Petri dish (**Figure 17**). The graphite rods are bound to two copper wires, which go through two small holes on the lid of the Petri dish. The holes and wires are sealed together with Loctite® Super glue.

The electrical stimulation setup consists of this stimulation device, an electrical pulse generator (we use Aim TTi TG2512A), and an oscilloscope. The pulse generator is connected to a T-adapter, which connects to the oscilloscope and to two positive and negative cables with alligator clips. The alligator clips are clipped to the wires on the electrodes.

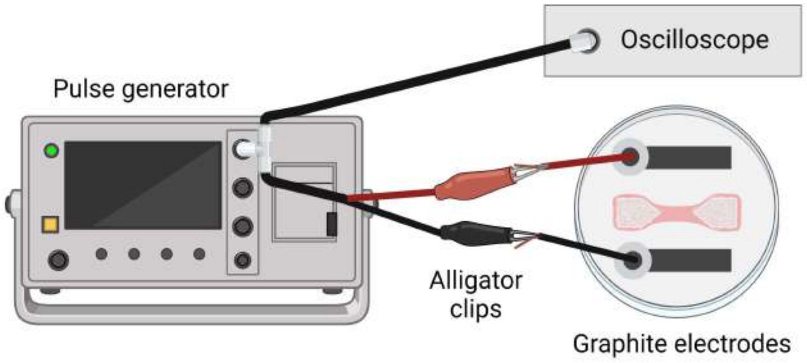

Figure 17. Electrical stimulation setup.

For additional details on the setup of the electrical stimulation equipment, see **Figure S7** in the associated PLOS One article.

19.2 On the day that stimulation is to be performed, if needed to maintain sterile conditions, **sterilize the stimulation device** by placing the lid onto a 35 mm Petri dish filled with 70% ethanol. Incubate for 00:15:00 , wash 3 times with sterile H_2O, and then sterilize the lid under UV light for 00:15:00 with the electrode part facing upwards. 30m

19.3 Inside the biological safety hood, **place the electrode lid onto the Petri dish** of interest, with the electrodes parallel to the muscle bundle (**Figure 17**). Take the closed plate to the microscope, and **fasten it to the microscope stage** using appropriate holder, or alternatively, two pieces of tape placed at the sides. This will avoid accidental opening of the lid when attaching the electrodes to the pulse generator.

For additional details on the setup of the samples for electrical stimulation, see **Figure S7** in the associated PLOS One article.

19.4 For stimulation, the electrodes on the lid are connected to a pulse generator, programmed with the following **recommended parameters**:

- Pulse duration: 10 ms
- Frequency: 1 Hz
- Amplitude: 10 V (monophasic)

Higher frequencies may be tested if it is desired to study the tetanization of the muscle fiber. Different voltages (5-20V) may be required depending on the cell line in order to see a robust contraction response. Pulse durations between 2-10 ms may also be tested.

The pulse generator is connected to an oscilloscope to verify that the pulses are correctly generated.

19.5 **Program the microscope settings.** Depending on the type of analysis that has been chosen (contraction analysis or calcium transient analysis), the microscope settings will be programmed differently. Go to **Step 33** or **Step 41**, respectively.

19.6 **Attach the graphite electrode cables to the pulse generator** using alligator clips, and fasten these cables to the microscope stage using a piece of tape, to avoid unwanted movement of the cables.

19.7 **Focus on the muscle bundle** at 20X magnification, in brightfield. It is recommended to focus on the edge of the bundle, as shown in **Figure 18**, to obtain better contraction images.

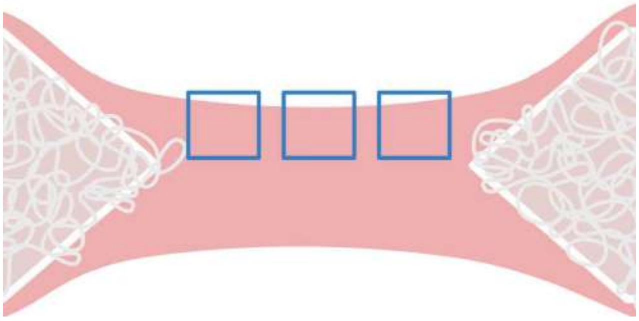

Figure 18. Best regions to focus on when recording stimulation videos.

19.8 When all the setup is complete, **start the video recording**. Apply the stimulation regimen shown in **Figure 19**: After 00:00:10 with no stimulation, turn on the pulse generator and stimulate the muscle bundle for the remaining time of recording (00:00:50). Then, turn off the pulse generator, unfasten the alligator clips, and return the cells to the incubator. Repeat the process with any remaining plates. 1m

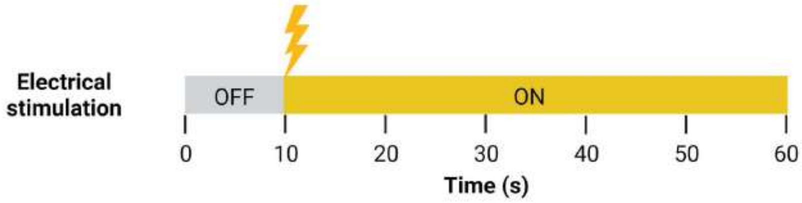

Figure 19. Electrical stimulation regimen during a 1-minute recording.

Preparation of solutions for chemical stimulation

20 As a positive control of chemical stimulation, prepare a solution of [M] 1 Molarity (M) **KCl in Milli-Q^{TM} H_2O.** It is not necessary to keep this solution sterile, although it may be filtered if desired.

21 On the day of stimulation, **prepare a stock solution of acetylcholine** in phenol red-free DMEM. Weigh the appropriate amount of acetylcholine chloride to prepare a 100X solution ([M] 10 millimolar (mM)). For example, for 10 mL of stock solution, weigh 18.2 mg of Acetylcholine chloride **Sigma Aldrich Catalog #A6625** and add 10 mL of phenol red-free DMEM DMEM, high glucose, HEPES, no phenol red **Thermo Fisher Catalog #21063029** . Finally, filter the solution with a 0.22 µm filter and store in the fridge until use.

> It is best to prepare the acetylcholine solution fresh, on the day that stimulation will be performed, to avoid degradation.

Chemical stimulation with acetylcholine and KCl

22 Before stimulation with chemical solutions, **ensure that the volume inside the Petri dish is 3 ml**. If it is lower, change/add medium before stimulation. Medium may have experienced evaporation in the incubator, which can affect the final concentration of the added solutions.

23 Turn on the microscope. Make sure to place both chemical solutions (ACh and KCl) next to the microscope. Bring pipettes and pipette tips in order to add the chemical solutions when needed.

If the plate contains 3 ml of medium, the amounts to be added of each solution are:

- **ACh**: 1:100 dilution --> 30 µl --> P100 pipette
- **KCl**: 1:10 dilution --> 300 µl --> P1000 pipette

24 **Program the microscope settings.** Depending on the type of analysis that has been chosen (contraction analysis or calcium transient analysis), the microscope settings will be programmed differently. Go to **Step 33** or **Step 41**, respectively.

25 Place the plate at the fluorescence microscope, and focus on the muscle bundle at 20X magnification. It is recommended to focus on the exterior part of the bundle (as in **Figure 18**) which will be more exposed to the chemical stimulant.

Focus with the lid taken off of the plate, to allow for quick addition of stimulation solutions. This means that the plate will no longer be sterile after stimulation.

26 **Start the video recording** and **perform the stimulation** according to the chosen stimulation regimen. In our experience, the stimulation regimens shown in **Figure 20** and **Figure 21** have been appropriate for quantifying the response to the applied stimulation, but may be modified for different needs.

At the chosen times, add the chemical solutions to the medium next to the recorded region, making sure to avoid disturbing the image. For this, pipette quickly (accurate start time of stimulation) but steadily (minimize vibrations in the medium and muscle construct).

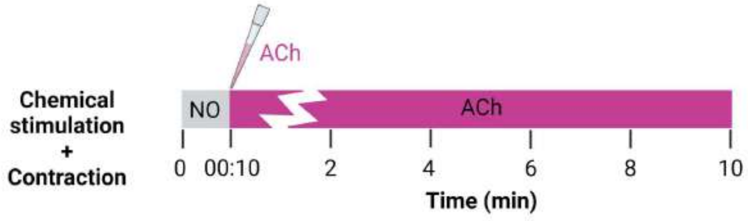

Figure 20. Example chemical stimulation regimen for contraction analysis in brightfield videos.

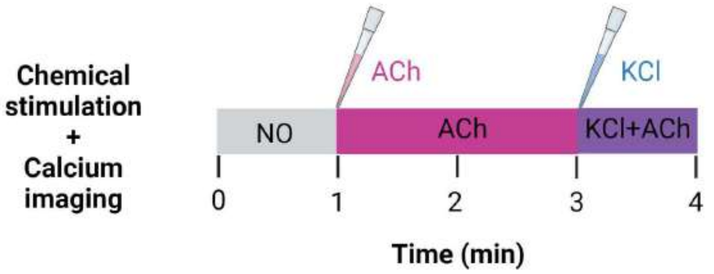

Figure 21. Example chemical stimulation regimen for calcium analysis with Fluo-4 AM and fluorescence microscopy.

Optical stimulation of ChR2+ cells

27 For optical stimulation, it is necessary to use a myoblast cell line which has previously been made to express channelrhodopsin-2 (ChR2). We have generated ChR2+ human myoblasts by viral infection and FACS purification, as described in a previous publication from our group:

Sala-Jarque J, Mesquida-Veny F, Badiola-Mateos M, Samitier J, Hervera A, Del Río JA (2020). Neuromuscular Activity Induces Paracrine Signaling and Triggers Axonal Regrowth after Injury in Microfluidic Lab-On-Chip Devices.. Cells.
https://doi.org/pii:E302.10.3390/cells9020302

After 14 days in differentiation medium, cells expressing ChR2 can be optically stimulated. Contractions are imaged in brightfield and processed using the MUSCLEMOTION plugin for ImageJ.

28 The optical stimulation setup consists of a coolLED pE-300 illumination system coupled to an inverted microscope (i.e., Olympus IX71) to visualize the muscle contraction. The TTL control of the illumination system allows for the delivery of precisely timed sequences of light. The TTL can be directed to an Arduino-UNO™ microcontroller pulse generator or alternatively a PulserPlus generator and Pulser v3.1 software (Prizmatix, Israel). The average light intensity with this setup is ≈ 20-25 W/cm^2 , measured at the culture dish with a Newport 1919 optical power meter (Newport Photonics, USA).

The two alternative setups are shown in **Figure 22** and **Figure 23**, respectively.

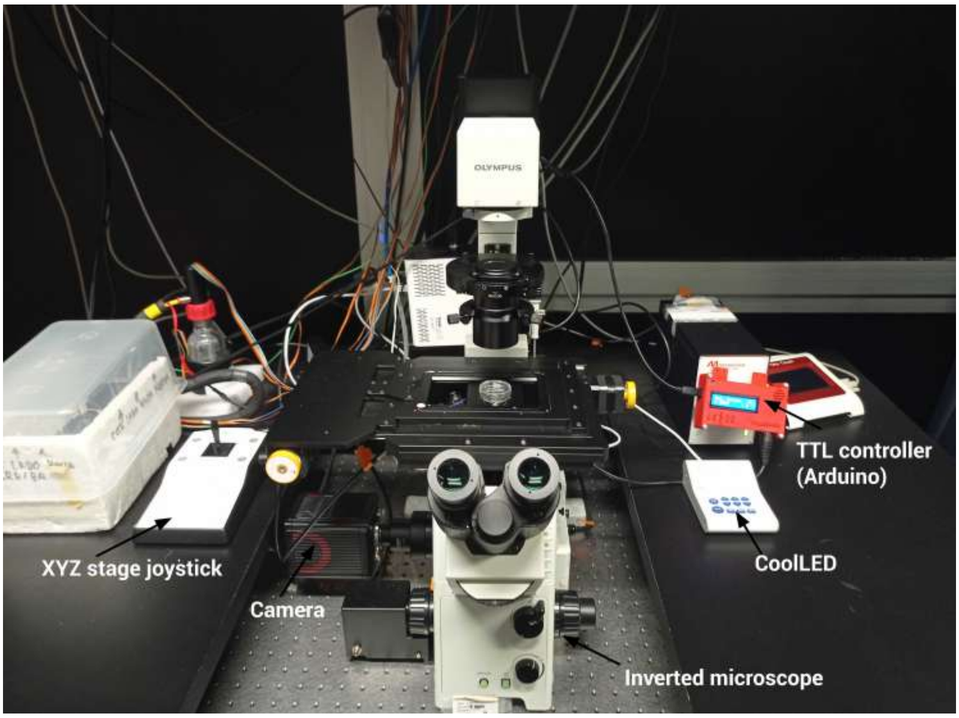

Figure 22. Optogenetic stimulation setup with Arduino-UNO™ microcontroller pulse generator.

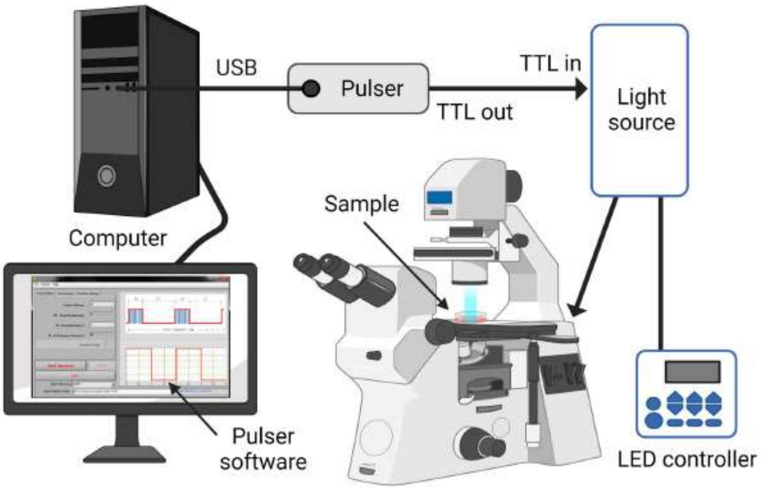

Figure 23. Optogenetic stimulation setup with PulserPlus generator and Pulser v3.1 software (Prizmatix, Israel).

29 For optical stimulation, turn on the Arduino or the pulse generator, and program the following **recommended parameters**:

- Pulse duration: 5 ms
- Frequency: 20 Hz
- Train duration: 1 s ON and 1 s OFF. (During the 1s when the stimulation is ON, 20 pulses/s are applied).
- Total duration: 1 min (30 repetitions of 1s ON + 1s OFF).

30 Program the microscope software to record a brightfield video with the parameters described in **Step 33**.

31 Turn on the bright field microscope illumination. Set up the image capture in live view. Fine-tune imaging positions and focus using the microscope software before starting the experiment. Use a microscope objective which allows the observation of individual cells (20X in our experiments).

32 When all the setup is complete, start the video recording. Follow the stimulation regimen shown in **Figure 24**: After 00:00:10 with no stimulation, turn on either the Arduino or the pulse generator and stimulate the muscle bundle for the remaining time of recording (00:00:50). Then, turn off the pulse generator and return the cells to the incubator. Repeat the process with any remaining plates. 1m

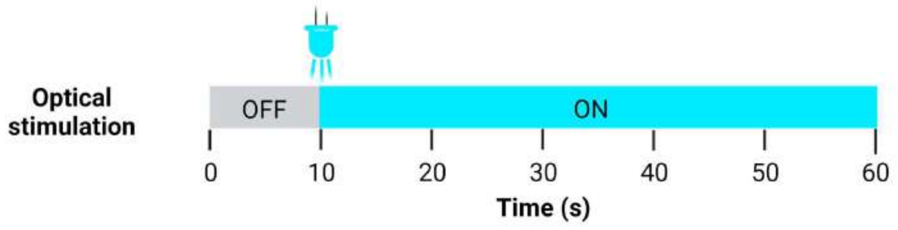

Figure 24. Optical stimulation regimen.

Contraction analysis with MUSCLEMOTION

33 If performing contraction analysis, program the following **recommended microscope settings for brightfield videos**:

- Resolution: 1024x1024 px, 16-bit
- Total time of recording: 1 min or more, depending on chosen stimulation regimen
- Frame speed: 40 fps (1 image every 25 ms)
- Exposure time: lower than 25 ms, check that the image acquisition time is faster than the desired frame speed.

Regarding software, commercial (such as Olympus cellSens) or open-source software (µManager) can be used to program these parameters.

34 After brightfield imaging of contraction, **analyze the contraction response with the MUSCLEMOTION plugin for ImageJ**. This protocol can be used for electrical, chemical or optical stimulation videos.

34.1 If the videos are obtained in certain microscope software formats such as .vsi, it will be necessary to open them with the Bioformats plugin (Bioformats Importer), and then save them as a .tif "Image Sequence". Opening and converting large videos may be time-consuming depending on your computer's processing speed.

34.2 Open the MuscleMotion plugin in ImageJ and click "Run". Select the frame rate according to the frame rate in the input video.
Leave all other parameters as shown in **Figure 25** and click OK. This will open a file selection menu to select the output directory (recommended to create a new folder) and the input directory (the folder with the TIFF image sequence).

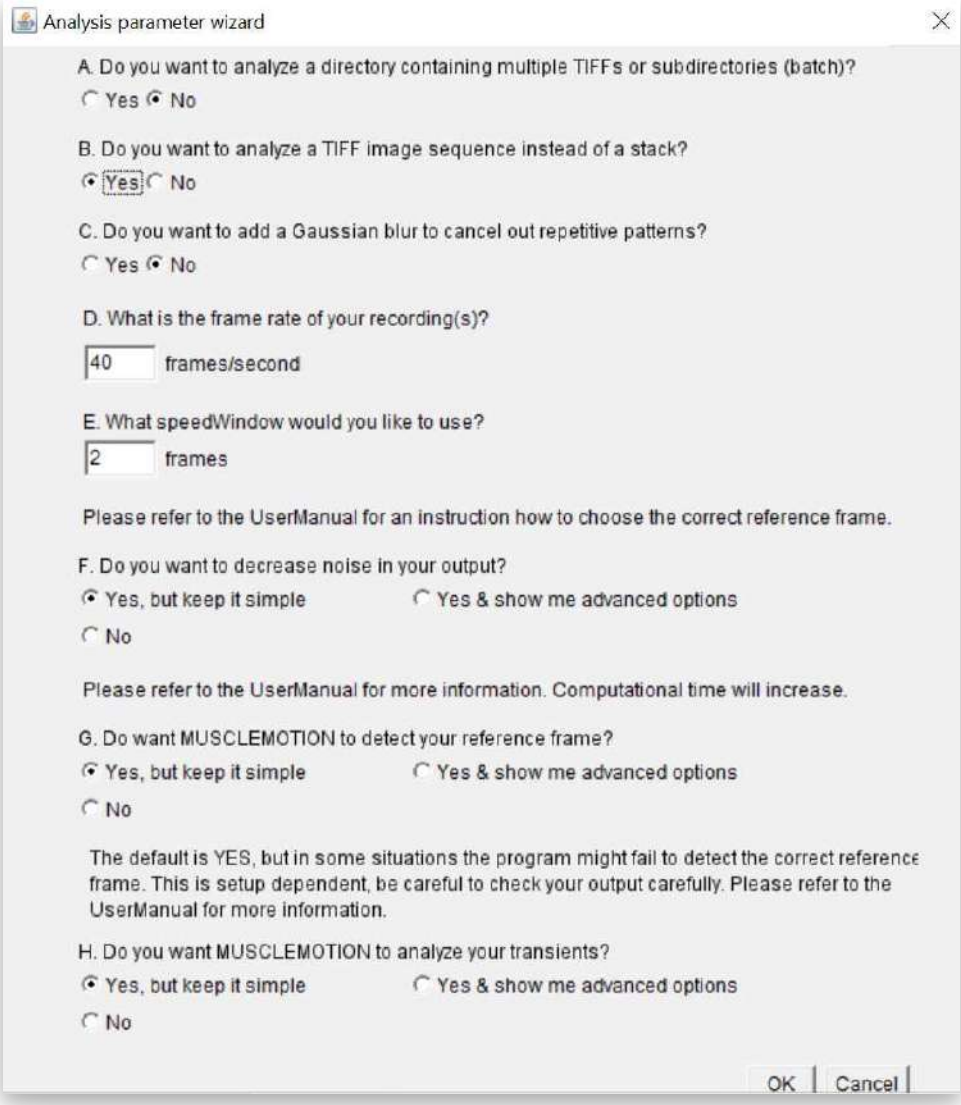

Figure 25. MuscleMotion parameter menu.

34.3 After running the program, the processing may take several minutes to be completed. The files that will be obtained in the output folder are:

- Reference frame
- Contraction: graph and text file
- Contraction speed: graph and text file

The text files contain contraction or contraction speed information at each frame, which can be used for creating a graph in any other software of your choice (for example, GraphPad Prism, as in **Figure 26**).

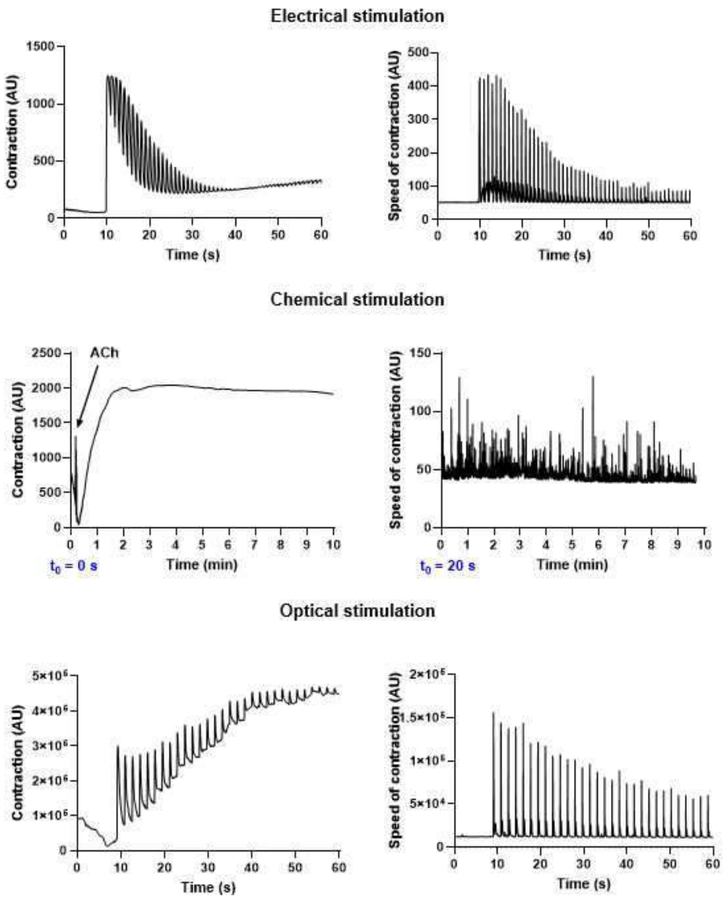

Figure 26. Examples of contraction and speed of contraction graphs obtained with different modes of stimulation. Graphs created in GraphPad Prism.

Calcium imaging: Fluo-4 AM loading and deesterification

35 Calcium imaging may be combined with electrical or chemical stimulation protocols.

Fluo-4 cannot be used as a calcium indicator in combination with ChR2^{+} cell lines, as both are excited with the same wavelength (blue light). If it is desired to combine calcium imaging with optogenetic stimulation, a genetically encoded calcium indicator with red emission spectrum (RCaMP) may be combined with ChR2. In this way, cells are optically stimulated with blue light and calcium imaging is performed using a red light filter.

36 **Fluo-4 AM sterile stock solution** should be prepared in advance at [M] **1 millimolar (mM)** in DMSO with [M] **20 Mass / % volume** Pluronic®, and stored at -20°C protected from light. 20m

To prepare the solvent, dissolve **2 g** of Pluronic® in **10 mL** of sterile DMSO Dimethyl sulfoxide (DMSO) **Sigma Aldrich Catalog #D2650** . To increase solubility, leave the mixture in a **37 °C** water bath for **00:20:00** min and vortex the solution until there are no more clumps of Pluronic®. Then, filter this mixture at 0.22 µm in the biological safety hood.

To reconstitute Fluo-4 AM, follow the manufacturer's instructions.

Fluo-4, AM, cell permeant **Thermo Fisher Catalog #F14217**

37 **Wash cells three times** with pre-warmed phenol red-free DMEM (DMEM, high glucose, HEPES, no phenol red **Thermo Fisher Catalog #21063029**).

38 1h

Add Fluo-4 AM at a final concentration of [M] **10 micromolar (µM)** in pre-warmed phenol red-free DMEM.

For each Petri dish, use **2 mL** of medium (enough to cover the muscle bundle) and **20 µL** of Fluo-4 AM stock solution ([M] **1 millimolar (mM)** in DMSO with [M] **20 Mass / % volume** Pluronic®). Perform loading of the Fluo-4 AM in the incubator for **01:00:00** .

39 30m

After loading is completed, . Then, for **deesterification of Fluo-4**, leave cells in phenol red-free DMEM at **Room temperature** for **00:15:00** and then at **37 °C** for another **00:15:00** .

40 **Proceed to imaging shortly after deesterification**. Fluorescence intensity and cell viability decrease after prolonged times in the incubator. Therefore, if imaging many samples on the same day, it is necessary to perform loading and imaging in a staggered manner.

41 **Recommended fluorescence microscope settings**:

- Total video time: depending on the type and regimen of stimulation.
- Magnification: 20X
- Fluorescence: ~70-80s exposure time, blue light (adjust for your microscope/lamp/cells). Must be lower than the acquisition time.
- Acquisition time: 100ms (10 fps).

Using an LED source is more appropriate for performing calcium imaging, as the light intensity remains more stable during recording than it is when using a halogen lamp.

Calcium transient analysis

42 In order to analyze videos obtained from Fluo-4 AM calcium imaging experiments, we use **ImageJ for processing and obtaining $\Delta F/F_0$ data**. In order to analyze calcium transients, individual cells are manually delimited using the ImageJ ROI manager. Then, fluorescence intensity data is extracted at each timepoint. This data is normalized to the mean basal fluorescence.

As an example, a video using chemical stimulation with [M]100 micromolar (µM) ACh and [M]100 millimolar (mM) KCl is analyzed in the following steps. This video was recorded at 100 ms/frame (10 fps), for a total of 4 minutes.

43 First, **open the video file in ImageJ**. Play the video file using the sliding bar, and identify the timepoint or frame at which the stimulation was initiated.

For example, in the case of a video with the stimulation regimen shown in **Figure 21** (with addition of acetylcholine and KCl), key timepoints would be 1 min (60000 ms = 600 frames) for ACh addition and 3 min (180000 ms = 1800 frames) for KCl addition, respectively.

By moving the sliding bar back and forth, visually identify cells that respond to the stimulation after the start of stimulation (see differences between **Figure 27** and **Figure 28**). These cells will later be selected as regions of interest (ROIs), as explained in the following steps.

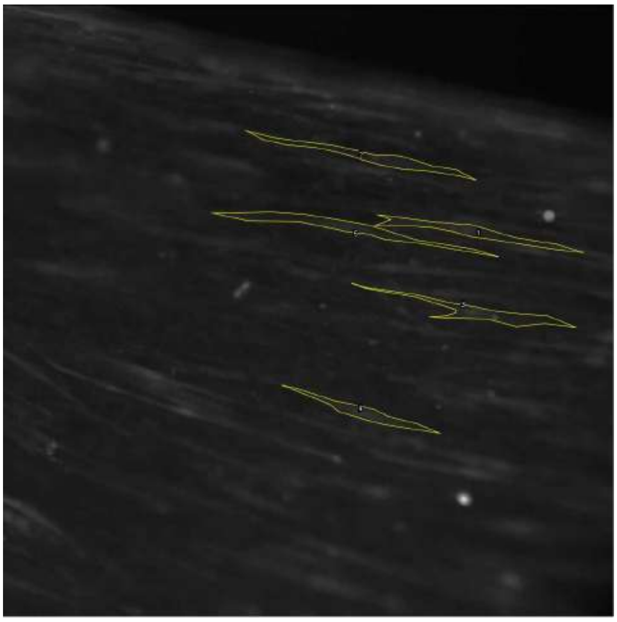

Figure 27. Five highlighted cells before any stimulation.

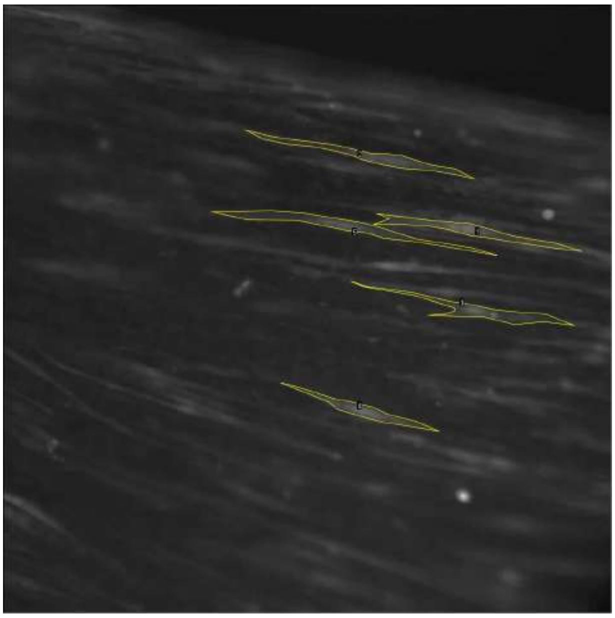

Figure 28. Five highlighted cells showing fluorescence increase after ACh stimulation.

44 After visually identifying the cells, proceed to **define ROIs of a subset of cells** (5-10 cells per video) and calculate their **fluorescence intensity over time**.

44.1 On the ImageJ toolbar, open the ROI manager. Go to Analyze > Tools > ROI manager.

44.2 Using the polygon selection tool , outline each of the cells of interest as in **Figure 29**. After closing each outline, on the ROI manager, click on "Add" before outlining the next cell. To see all the ROIs, click on "Show all" At the end of the selection, the ROI set can be saved using More >> Save.

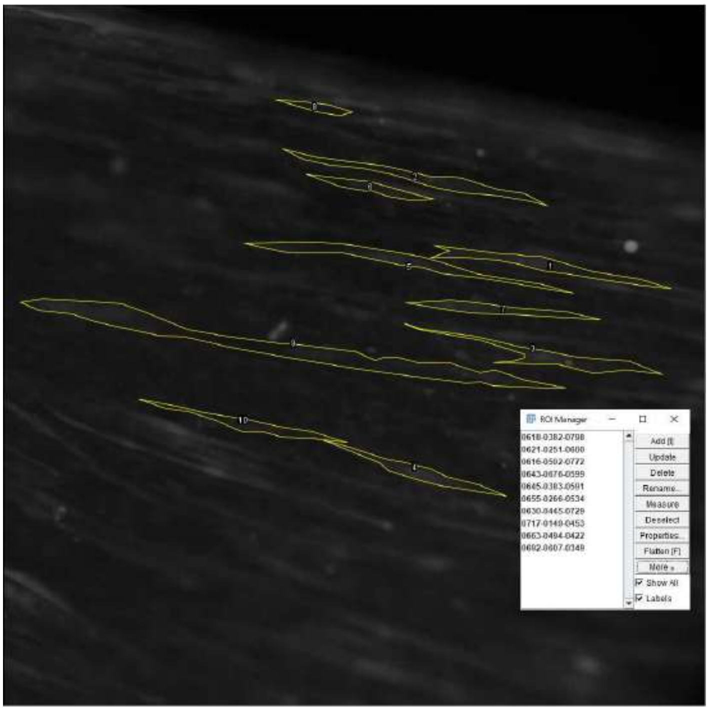

Figure 29. Example of 10 ROIs selected with the ImageJ ROI manager.

44.3 Once the ROIs are selected and saved, go to Analyze > Set Measurements and select "Mean gray value" only (**Figure 30**). In this way, only the fluorescence information will be extracted from the ROIs. Click OK.

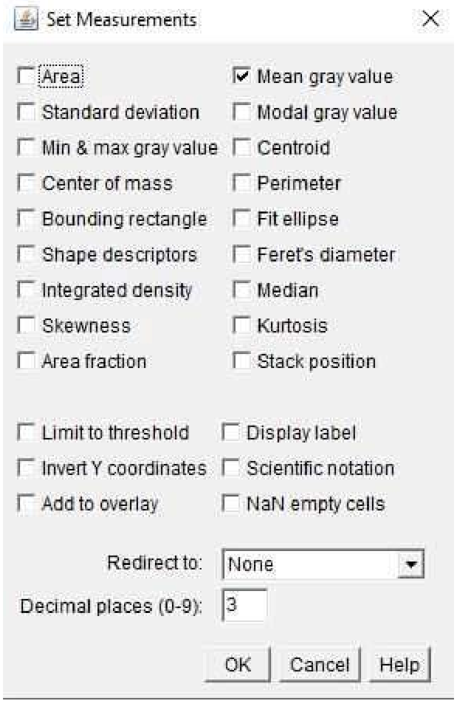

Figure 30. "Set measurements" window.

44.4 Then, using the ROI manager, click on More >> Multi Measure to measure mean gray value of all ROIs at each timepoint (**Figure 31**). Enable the first two options on the window that pops up (**Figure 32**).

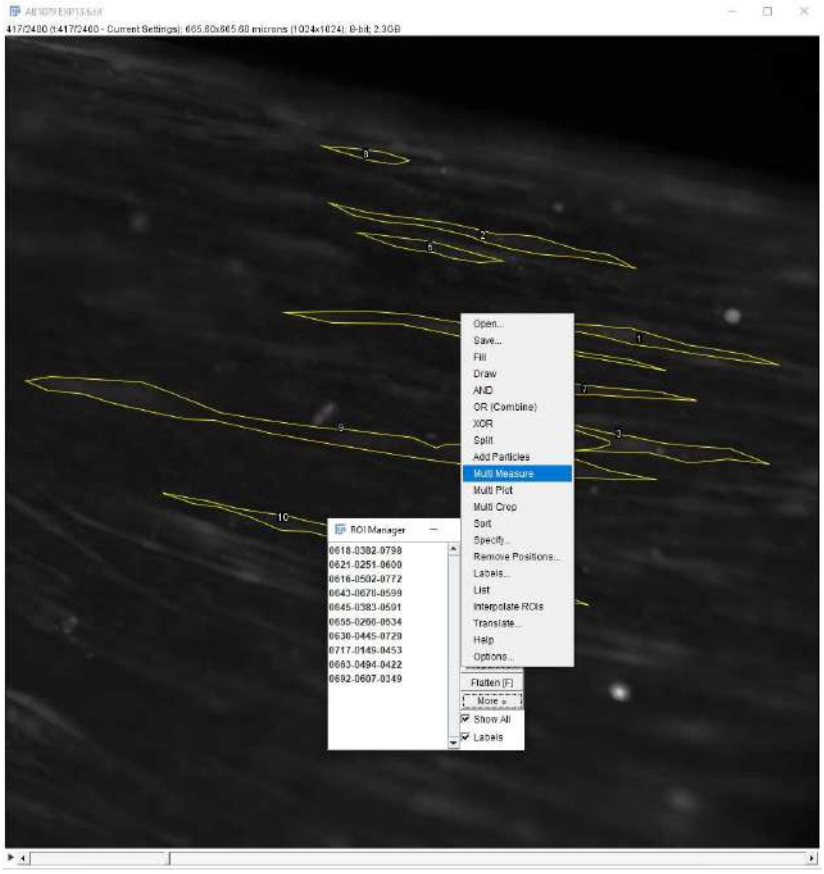

Figure 31. Multi Measure option.

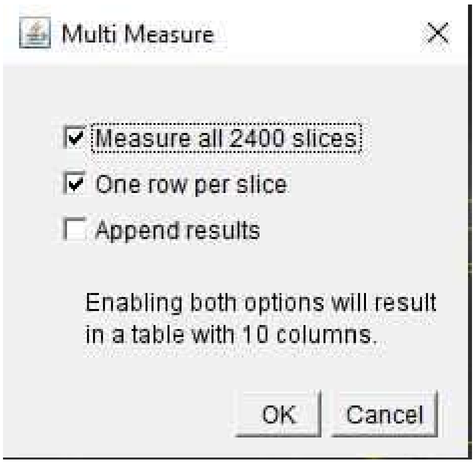

Figure 32. Multi Measure parameters.

44.5 After the measurements are completed, the results will pop up in a Results window. Each column contains the fluorescence intensity data for one ROI at all the timepoints (each row is one frame). Save these results and/or copy them to an Excel file (or other similar spreadsheet software).

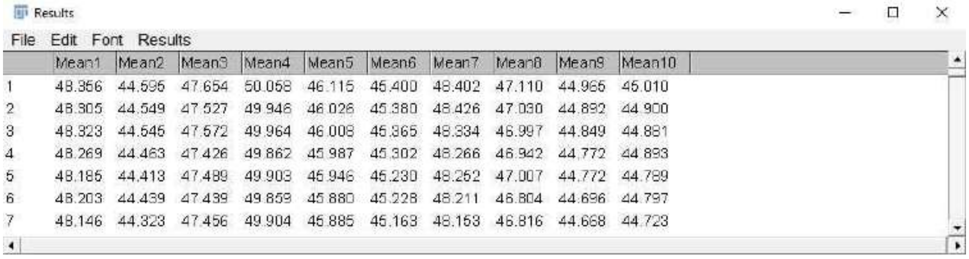

	Mean1	Mean2	Mean3	Mean4	Mean5	Mean6	Mean7	Mean8	Mean9	Mean10
1	48.356	44.595	47.654	50.058	46.115	45.400	48.402	47.110	44.965	45.010
2	48.305	44.549	47.527	49.946	46.026	45.380	48.426	47.030	44.892	44.900
3	48.323	44.545	47.572	49.964	46.008	45.365	48.334	46.997	44.849	44.881
4	48.269	44.463	47.426	49.862	45.987	45.302	48.266	46.942	44.772	44.893
5	48.185	44.413	47.489	49.903	45.946	45.230	48.252	47.007	44.772	44.789
6	48.203	44.439	47.439	49.859	45.880	45.228	48.211	46.804	44.696	44.797
7	48.146	44.323	47.456	49.904	45.885	45.163	48.153	46.816	44.668	44.723

Figure 33. Results window after multi measure.

45 **Normalize the fluorescence data to the basal fluorescence (F_0)** in order to **calculate $\Delta F/F_0$.**

45.1 With the video still open, go to Image > Stacks > Z project...

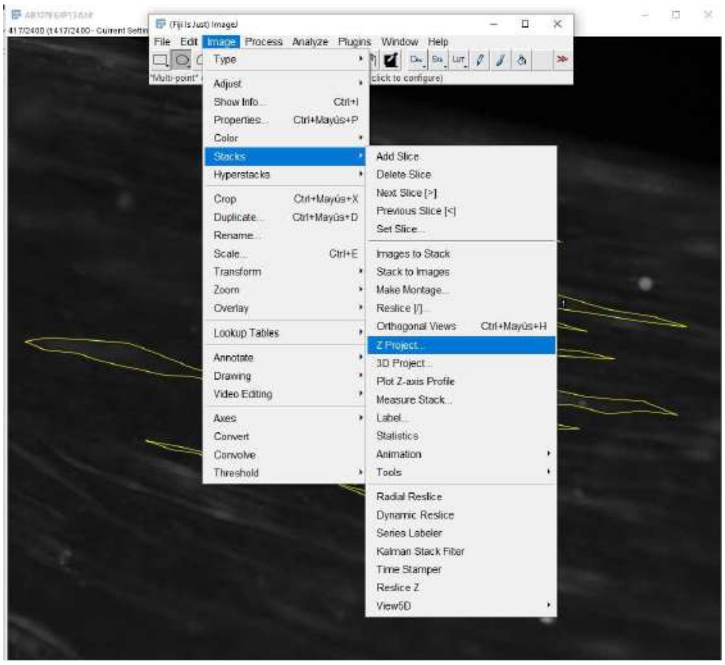

Figure 34. Image > Stacks > Z project...

Select the projection type, which is "Average Intensity", and click OK.

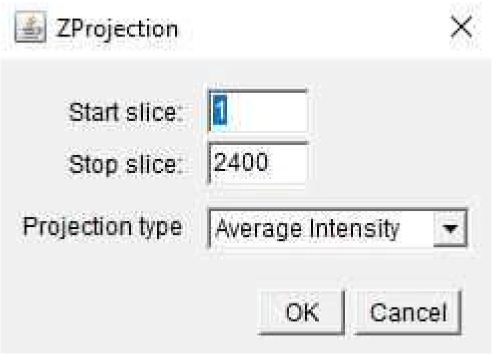

Figure 35. ZProjection window.

45.2 After this, a Z projection of the video will pop up. In this image, select a ROI containing only the background, which can be any shape (in **Figure 36**, an ellipse was used).

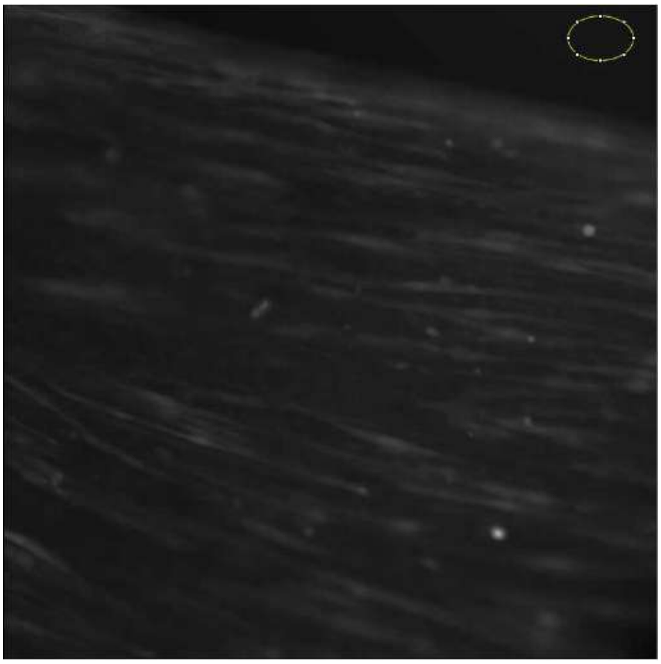

Figure 36. Ellipse ROI in top right corner, containing only background.

45.3 While this ROI is selected, go to Analyze > Measure in order to obtain the fluorescence value for this ROI. Save the result that pops up (this will be $\mathbf{F_0}$).

45.4 Using Excel or another spreadsheet software of your choice, normalize the fluorescence data from each ROI to the $\mathbf{F_0}$ as shown in **Figure 37**.

$$\frac{\Delta F}{F_0} = \frac{F - F_0}{F_0}$$

Figure 37. Formula for calculation of normalized fluorescence.

46 Use a software of your choice to generate an XY plot of the $\mathbf{\Delta F/F_0}$ over time. In our case, we use GraphPad Prism software, but other open-source software such as Veusz can be used instead.

- X values: time, in increments of 100 ms, or as many ms as separate each frame.
- Y values: $\Delta F/F_0$ of each ROI (plot all ROIs in the same graph).

In order to visualize the point at which the stimulation was applied, arrows can be added as annotations. It is also helpful to show each ROI in a different line color.

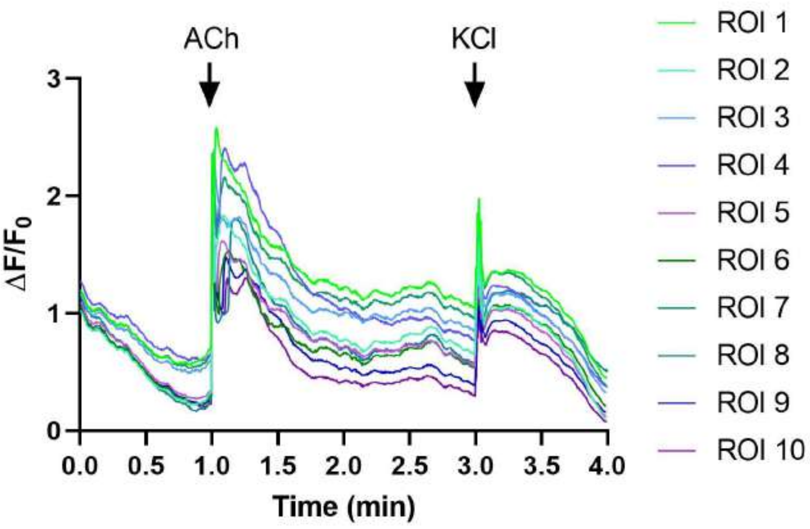

Figure 38. Graph of $\Delta F/F_0$ over time in cells stimulated with ACh and KCl. Created with GraphPad Prism software.

After these steps, the data generated with this protocol can be used in further analyses that may be interesting for each researcher's purposes.
