# Supplementary figures and images for: Development of a simple and versatile in vitro method for production, stimulation, and analysis of bioengineered muscle
Source: PLoS One. 2022 Aug 11;17(8):e0272610. doi: 10.1371/journal.pone.0272610 (PMC9371355; doi:10.1371/journal.pone.0272610)

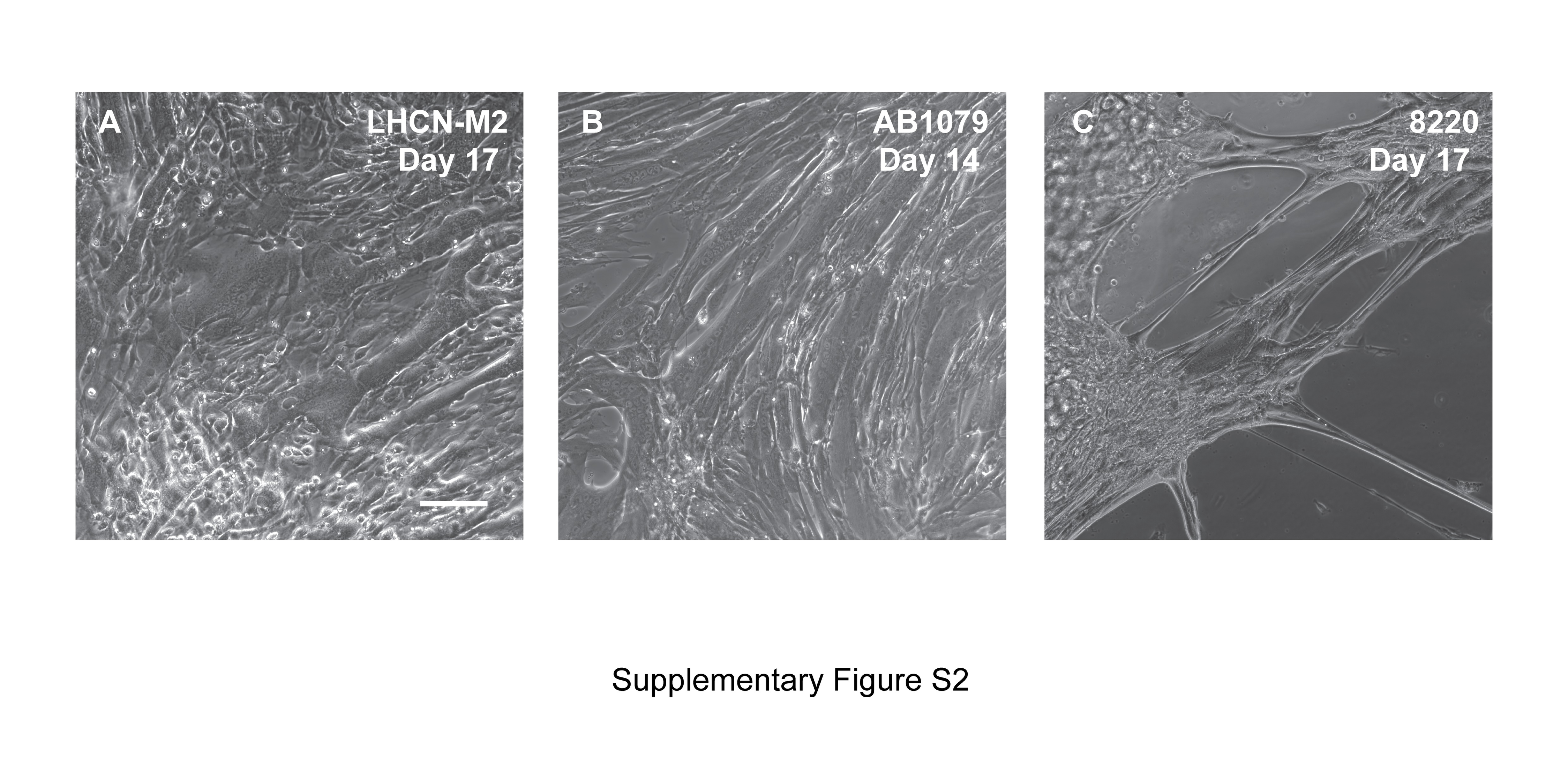

Supplement: S1 Fig — Phase contrast photomicrographs of three myoblast cell lines (LHCN-M2, AB1079, and 8220) differentiated for 14–17 days on gelatin-coated coverslips. To avoid myotube detachment, cells were covered with an overlay of MatrigelTM Growth Factor Reduced (GFR) Basement Membrane Matrix (1:3 dilution in DMEM). Note the lack of clear alignment between myotubes. Scale bars: A = 100 μm pertains to B-C. (TIF) [file pone.0272610.s002.tif]

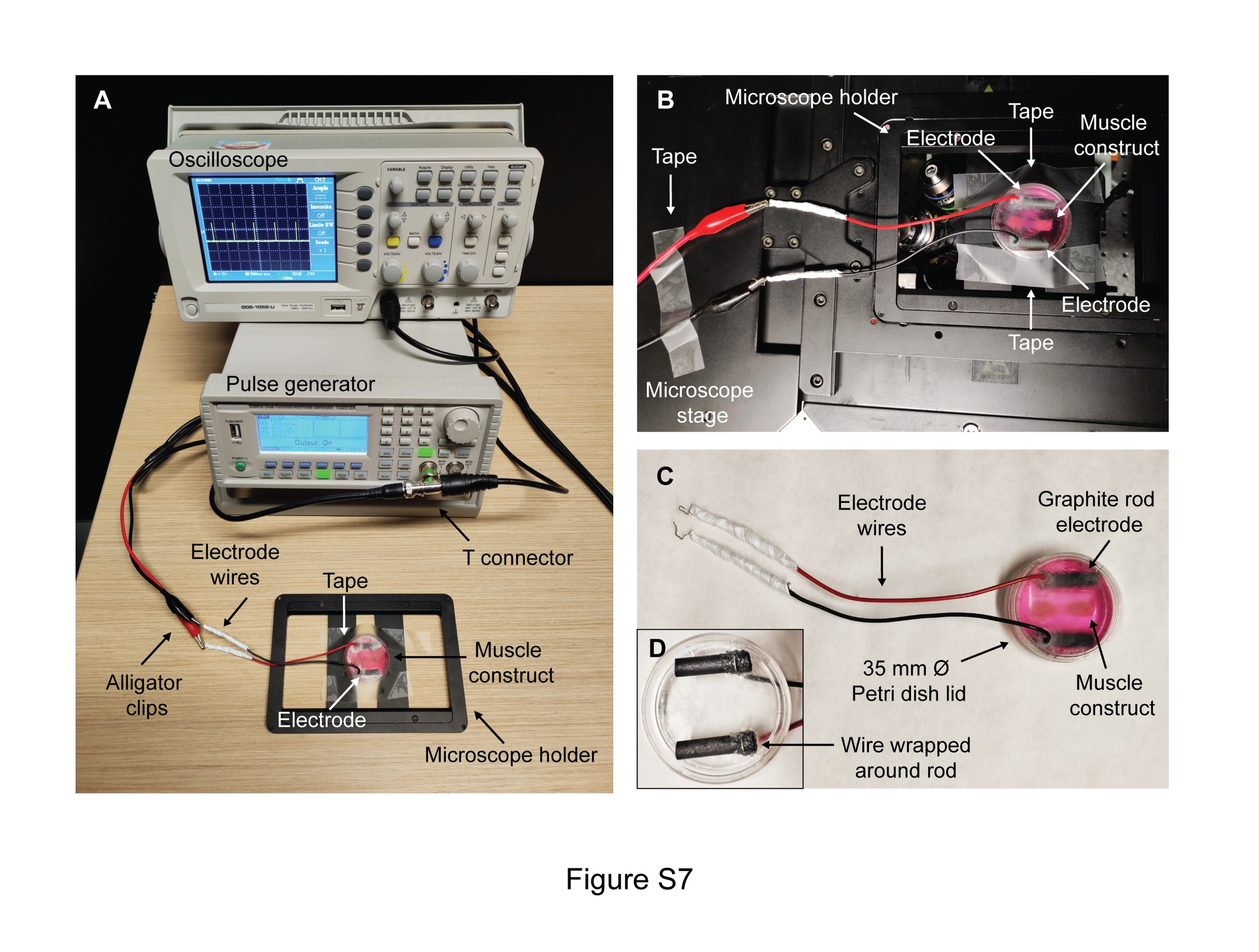

Supplement: S2 Fig — A) Overall view of stimulation equipment and setup. The pulse generator is connected to the electrodes via a connection to a pair of alligator clips, and also to an oscilloscope (use a T connector to achieve this dual connection). B) Placement and fastening of the sample to the microscope stage. Tape is placed on the plate to fasten the lid to the microscope holder, and also on the alligator clips to avoid movement during focusing. The electrodes must remain parallel to the construct. C) Close-up of the stimulation device placed on a muscle construct. See how the wires are wrapped around the graphite rods in D). Wires are sealed to the lid and kept in place with Loctite® Super Glue. (TIF) [file pone.0272610.s003.tif]
